# Effects of differential contacts with the criminal legal system on mental health outcomes of adolescents and young adults: A fixed-effects model

**DOI:** 10.1371/journal.pone.0344895

**Published:** 2026-06-17

**Authors:** Raquel V. Oliveira, Elizabeth Culatta

**Affiliations:** 1 Department of Social Sciences, Pamplin College, Augusta University, Augusta, GeorgiaUnited States of America; 2 Analyses and Metric Validation Research Group (CNPQ – UNESP), Universidade de São Paulo, Araraquara, Brazil; The Hong Kong Polytechnic University, HONG KONG

## Abstract

Research has indicated that contacts with the criminal legal system may have detrimental consequences for health and well-being. There is, however, a lack of research exploring how different types of interactions with the criminal legal system may impact mental health. Our study aims to address the gap in the literature by exploring progressively more severe forms of criminal legal contact on a variety of mental health symptoms using the Pathways to Desistance Study (PDS), a longitudinal sample of youth (2000–2010) who have a high level of contact with the criminal legal system (N = 1,322). Using fixed-effects modeling, we examine how specific types of contact with the criminal legal system (e.g., arrests, court appearances, institutionalization) are associated with mental health outcomes including symptoms of anxiety, depression, hostility, and psychoticism. We found that arrests and institutionalization have significant cross-sectional associations with multiple mental health symptoms, while appearing in court was associated with more long-term increases in reported mental health symptoms. We also observed that substance use, criminal involvement, and use of mental health medication were consistently associated with mental health outcomes across 10 waves of data. Thus, our paper evidences that even less severe contacts with the criminal legal system appear to be stressors that can have both short-term and long-term effects on mental health symptoms in youths and young adults.

## Introduction

There is an overrepresentation of individuals with mental health issues in the criminal legal system when compared to the general population, with 43% of state and 23% of federal prisoners in the United States reporting a history of a mental health problem [1]. Due to this overrepresentation of mental health diagnoses in individuals involved in the legal system, there have been many reports associating mental illness with increased risk of engaging in illegal behaviors [2–6]. However, another body of research has suggested that contacts with the criminal legal system can increase risk for mental illness, substance use disorders, and exacerbate mental health symptoms [7–11].

Within this body of research, scholars have considered different forms of contact with the criminal legal system. Specifically, studies have focused on the detrimental mental health outcomes of incarceration [11,12] and of initial police contact on mental health outcomes [13,14]. Further, recent studies, discussed below, have also explored a continuum of contact with the criminal legal system on health outcomes [15–17]. This literature has, for the most part, focused on mental health outcomes such as anxiety [18–20] and/or depression [14,15,19].

Extending this line of research, in this article we examine the effects of different types of criminal legal contacts, including arrest, court appearance, and institutionalization (which includes being held in a secure institution, locked facility, jail, detention, prison, or residential treatment center) on a range of mental health outcomes. Specifically, using a longitudinal sample of high-risk youths and emerging adults, we examine the effect of being arrested, being called to appear in court, and being institutionalized (including facilities designed for minors) on mental health outcomes, such as hostility and psychoticism in addition to anxiety and depression. We explore if negative mental health symptoms are associated with contacts with the criminal legal system by using a fixed-effects approach in both cross-sectional and longitudinal models.

### Mental health and the criminal legal system

There is an overrepresentation of individuals with mental health issues in the U.S. criminal legal system. Reports have indicated that while 22.8% of adults in the general population have been diagnosed with any mental illness, between 30–40% of persons incarcerated in jails and prisons have reported having some mental illness diagnosis with 13–26% meeting the criteria for serious psychological distress [1,21–23]. The most common mental health disorders reported in legally-involved adult populations, excluding substance use disorders, are depression (25–30%), anxiety (10–22%) and bipolar disorder (9–25%), with psychotic disorders also having a high representation in these populations (3–11%) [24–27]. These values far exceed those of the adult general population (depression 8%, bipolar 3%, psychotic disorders <0.7%), with the exception of anxiety (19% for general population) [26].

Similar to adults, youths in the criminal legal system have consistently higher prevalence of mental health issues than those in the general population. Specifically, research has found 40–80% of incarcerated youths have a diagnosable condition compared to 15–22% of youths in the general population [28–30]. The most common mental health issues in legally-involved youth are depression (8–31%) and conduct disorder (45–73%), with psychotic disorder (2–3.5%) also being overrepresented in these groups [31].

This overrepresentation of mental illness among legally-involved individuals has led researchers to explore the complex interplay between mental health and crime. While some research has suggested there might be a relationship between specific mental health symptoms and criminal offending [32–36], a large body of research suggests that mental illness on its own does not cause crime [37–40]. Further, there seem to be several common risk factors for both participation in illegal activities and developing mental health issues, including structural factors and exposure to several stressors.

Mental health symptoms and contact with the criminal legal system are thought to be related through three primary pathways. First, mental health symptoms increase the risk of criminal involvement, and this relationship may be mediated/moderated by other variables including substance use [41–46]. Second, there may be reverse causal order where contacts with the legal system are detrimental to mental health [16,18,47]. Finally, there might complex reciprocal effects between mental health issues and contacts with the criminal legal system [20,48]. We will briefly introduce each of these pathways here.

First, some research suggests that particular mental illness symptoms are linked to an increased risk of criminal participation [32–34,49,50]. In our study, in line with prior research [45,51,52], when referring to mental illnesses we are considering those classified as mood disorders (i.e., bipolar and related disorders, depressive disorders), anxiety disorders, and schizophrenia spectrum and other psychotic disorders. Substance use disorders and personality disorders are considered separate diagnoses and distinct from mental illnesses. Indeed, several studies explore variables that may mediate or moderate the relationship between mental illness and crime including substance use, personality disorders, and other social factors [51–55]. Research has shown that individuals with a mental illness diagnosis are at higher risk of having a comorbid substance use disorder [22,43,44]. Substance use disorders, in turn, have been associated with a higher likelihood of criminal involvement and recidivism [55,56]. Similar to mental illness symptoms, there is an overrepresentation of substance use disorders in legally-involved groups when compared to the general population. Some studies have indicated that substance use disorders are present in 50–75% of incarcerated adults [56,57] and 20–50% of legally-involved youths [30,58–60] compared to about 17.1% of the general population 12 + years old [22]. The presence of this comorbidity between mental health issues and substance use disorders has been linked to poor life outcomes, including an increased risk of criminal involvement, incarceration, and criminal recidivism [43,56].

Second,  this relationship between mental illness and criminal involvement may be explained by reverse causal ordering. Previous research links incarceration with negative mental health symptoms or health behaviors [8,61–63]. Further, recent research (explored in detail below) has highlighted the relationship between different forms of contact with the criminal legal system and mental health outcomes [15–17]. Specifically, this research explores the effect of being stopped by police, being arrested, receiving a conviction, and being incarcerated on symptoms of depression and anxiety [15–17].

Finally, some studies examine the reciprocal effects between these variables suggesting that contacts with the criminal legal system increase mental health symptoms, and at the same time having mental health issues may also increase the risk of contacts with the criminal legal system. Specifically, studies have reported that mental health symptoms are associated with arrest and incarceration rates, and that being arrested has effects on depression and anxiety symptoms [20,48,64–66].

### Criminal legal contact during adolescence and early adulthood

Life-course theories suggest that individuals’ behavior tends to follow particular age-graded patterns, with sources for continuity and change present at different life stages [67,68]. Specifically, life-course perspectives examine how particular life events can serve as “turning points” leading to changes in behavioral patterns. For instance, in the criminological context, these turning points can either lead to a desistance from crime (positive), or an increase in participation in crime (negative) [69]. Positive turning points divert individuals from criminal participation by promoting the development of social bonds with prosocial partners/peers or creating stakes in conformity. These frequently include conventional institutional ties of marriage, employment, or parenthood as adolescents grow into adult life-course transitions [70–72].

Research in general has found support for the effects of marriage, employment and parenthood as positive turning points [71,73–76]. It is important to note though that not all marriages or jobs lead to desistance, in fact some researchers have shown that the quality of the relationships and stability of employment are fundamental in reducing continued involvement in crime [77]. Similarly, parenthood seems to have stronger effects on women [78] especially when becoming a parent for the first time [75].

Research has also highlighted the importance of *negative* turning points when individuals become more involved with illegal activity [79]. Such negative turning points include events such as school suspensions and expulsions [80,81], gang involvement [82,83], or early criminal legal contacts [64–66,84]. All of these turning points have been consistently associated with a high risk of continued participation in criminal activities and recidivism.

Classic research on stress [85] explains how inequalities in mental health are directly related to social experiences (i.e., life events or chronic strains) that differ based on life circumstances and individuals’ resources to react to stressors. The Stress Process Model [86,87] explains that the social stress process contains three core elements: sources of stress (including contact with the criminal legal system), moderators of stress (not directly explored in this study), and manifestations of stress (both physical and mental). The sources of stress include any condition that arouses the adaptive capacity of the individual with an understanding that some individuals are disproportionally exposed to higher levels of stress based on their socioeconomic status, gender, or racial characteristics. Moderators include both social resources as well as personal resources. Finally, manifestations of stress include a range of physical and mental health outcomes. By identifying increased exposure to stressors and the level of moderating factors to mitigate or exacerbate the process, researchers can predict patterns of health outcomes for individuals [88]. Indeed, studies have used the social stress paradigm as the link between incarceration and physical health [89] and depression [12,90].

### Criminal legal contacts and mental health outcomes

#### Arrest and mental health.

While there is literature on initial police contact (such as stop-and-frisk) and mental health [e.g., 9,19], there is a dearth of research on other contacts with the criminal legal system, such as arrest and court appearance, on mental health. This may be partially because of the nature of these contacts – some individuals are arrested and released while others go on to experience a court proceeding, or even conviction and incarceration, so pinpointing the effects of arrest on later outcomes is difficult [91].

Despite the difficulty in isolating the effect of arrest, some research explores the consequences of arrest on general health and life outcomes with a special focus on minority groups overrepresented in arrest populations [7,92]. For example, a longitudinal study on health behavior using a sample of Black Americans finds an effect of having been arrested in young adulthood on smoking, daily drinking, and risky sexual behaviors in midlife for men, but not for women [92]. To the best of our knowledge, only a couple of studies have explored the relationship between arrest and mental health outcomes. Boen [15] finds that arrest is associated with symptoms of depression, net of other contacts with the criminal legal system, including being stopped by police, convicted, or incarcerated. However, some research contradicts the expected relationship between arrest and mental health, as Silver et al., [20] found no connection between the number of arrests and depression or anxiety. Given the lack of studies focused on arrest and the mixed findings reported, more research is needed on this topic.

#### Courts and mental health.

Most studies on the effects of court appearances on mental health outcomes have focused on victims, specifically on the process of secondary victimization by being exposed to criminal investigations and court proceedings [93–95]. Other research on family courts has also identified that children involved in court proceedings are more likely to experience depression and anxiety when compared to their counterparts who were not involved in proceedings [96]. However, very little research has been done exploring the detrimental health and mental health outcomes for defendants who go through court proceedings. To the best of our knowledge, only one prior cross-sectional study by Clemente and Padilla-Racero [93] explored the mental health outcomes of court appearances. The authors observed that defendants presented deteriorated mental health as an outcome of court proceedings, with greater deterioration occurring in longer proceedings. Some research has explored the detrimental outcomes of *convictions* [e.g., 16,17,97,98]; however, this is a fundamentally different measure as receiving a conviction implies having been found guilty and received a formal sanction, while when going to trial there is the presumption of innocence (“innocent until proven guilty”) and no formal sanction.

#### Incarceration and mental health.

Researchers have extensively explored the negative mental health outcomes associated with incarceration, especially among adults. Research focusing on young adults’ criminal legal contact found a higher probability of depression among individuals who experienced incarceration [14,15]. For example, Barnert et al. [8] examined a range of adult health outcomes, including depressive symptoms, and found that cumulative incarceration length during adolescence and early adulthood is independently associated with worse physical and mental health later in adulthood.

Further, studies have shown that interactions with correctional officers, medical staff, and other prisoners while incarcerated are *primary stressors*. Following institutionalization, *secondary stressors* can include difficulty in finding a source of income, trouble finding a place to live, being prohibited from political participation, or having restricted parental rights. While the primary stressor may be brief and limited, these secondary stressors may be ongoing with no definite endpoint [61,63,99]. Despite the substantial effects of incarceration of mental health symptoms, these researchers urge us to think of contacts with the criminal legal system as a continuum, where even the earliest stages of the process can detrimentally impact health and mental health outcomes.

#### Continuum of contact with criminal legal system.

Some authors have explored the outcomes of a continuum of contact with the criminal legal system, starting with contacts with the police, progressing to convictions and sentencing, and finally incarceration. For example, using the National Longitudinal Survey of Youth (NLSY) 1997, Sugie and Turney [16] examine the impact of arrest, conviction, and incarceration on a general measure of mental health. Based on their findings these authors suggest that even minor points of contact, such as arrest, can have deleterious mental health outcomes. In another study using the same dataset (NLSY97), Fernades [17] examines the effects of arrest, conviction, and jail sentences on both physical and mental health outcomes, finding that even less serious forms of contact negatively affect both forms of health. Using National Longitudinal Study of Adolescent to Adult Health data (Ad Health), Boen [15] examines the pre-incarceration contacts of being stopped by police, arrested, or convicted on outcomes of depressive risk and inflammation. The author finds that only arrest and incarceration have a direct negative association with the risk of depression. Taken together, these studies suggest the negative impact of contact with the criminal legal system on mental health can begin well before incarceration.

### Current study

The current study explores the relationship between differential criminal legal contacts and mental health outcomes, expanding upon prior research in three main ways. First, we add to previous literature by examining additional types of contact with the criminal legal system. Specifically, while most research examines the effects of police contact and incarceration, we also include arrests, court appearances, and institutionalization not limited to incarceration. Second, we examine different types of mental health outcomes, namely hostility and psychoticism, in addition to anxiety and depression. Finally, we estimate fixed-effects which allows us to control for time invariant factors, and dynamic fixed-effect models with lagged measures of criminal legal system contact on mental health outcomes at a later wave as Testa and colleagues [84] do in their work on future orientation. We examine the following two research questions: 1) is differential contact with the criminal legal system associated with mental health symptoms? and 2) does differential contact with the criminal legal system in one wave affect symptoms of mental health at the following wave?

## Methods

### Data and participants

We used data from the Pathways to Desistance Study (PDS; available from https://www.icpsr.umich.edu/web/NAHDAP/series/260 see details under Ethics Statement) which follows 1,354 adjudicated (i.e., the court concluded the juvenile committed the illegal act they have been charged with. Adjudication is not the equivalent of a conviction in adult criminal court [100].) youth from juvenile and adult courts in Phoenix (N = 654) and Philadelphia (N = 700) [101,102] as they transition from adolescence into early adulthood. These two locations were chosen due to (a) high rates of serious crime committed by youths; (b) racial/ethnic diversity of participant pool; (c) enough female serious offenders to allow to examine sex differences; (d) local support for the study and cooperation from the practitioners in the criminal and juvenile legal systems; and (d) the presence of experienced research collaborators to oversee the data collection [103]. This data has been used to explore longitudinal relationships in a sample of youth that had a high level of contact with the criminal legal system as they were found guilty of a felony offense at baseline [46,84].

The baseline interview was conducted when participants were ages 14–18 between November 2000 and January 2003, and 10 follow-up interviews were conducted over a 7-year period. By the last follow up interview participants were 21–25 years old. Youths were selected for potential enrollment based on their court files, with approximately one in three adolescents adjudicated for these charges being included in the study. The PDS researchers limited the proportion of male drug offenses to 15%, avoiding an overrepresentation of these offenses. This limitation was not applied to women given the common underrepresentation of females in legally-involved samples. Of the original 1,354 participants, 1,134 completed all 10 follow-up interviews representing an 83% retention rate by the last wave of data [101,103]. For our analysis we focused on all follow-up data (Waves 2–11).

### Measures

#### Dependent variables.

*Mental health.* We examine four separate dimensions of mental health: depression, anxiety, psychoticism, and hostility measured using items from the Brief Symptom Inventory (BSI; [104]). Each BSI dimension is measured using 5 or 6 primary symptoms in response to a question asking, “how much that problem has distressed or bothered you *during the past 7 days* including today.” The answers range from zero to four with 0 = “Not at all;” 1 = “A little bit;” 2 = “Moderately;” 3 = “Quite a bit;” and 4 = “Extremely.” The pattern for each of the four mental health symptoms was that higher rates were reported in earlier waves with hostility as the most reported issue (with 75% of the respondents reporting some symptoms of hostility in Waves 1–2).

*Depression.* Respondents were asked to report if, in the past 7 days, they had experienced 6 symptoms. These symptoms include feeling lonely or blue, feeling hopeless about the future, or having thoughts of ending their life. Across all waves, between 46–70% of respondents reported at least some symptoms of depression with higher percentages occurring in earlier waves.

*Anxiety.* Respondents were asked if, in the past 7 days, they had experienced 6 symptoms. These symptoms include being suddenly scared for no reason, nervousness or shakiness inside, or feeling tense or keyed up. Across all waves, between 51–64% of respondents reported at least some symptoms of anxiety with higher percentages occurring in earlier waves.

*Psychoticism.* Respondents were asked to report if, in the past 7 days, they had experienced 5 symptoms such as feeling lonely even when you are with other people, or agreement with the idea that someone else can control your thoughts, or the idea that something is wrong with your mind. Across all waves, between 43–66% of respondents reported at least some symptoms of psychoticism with higher percentages occurring in earlier waves. With the exception of Waves 1 and 2, when symptoms of anxiety were reported by slightly fewer respondents, symptoms of psychoticism were consistently reported the lowest of these four measures of mental health throughout the 10 waves.

*Hostility.* Respondents were asked to report if, in the past 7 days, they had experienced 5 symptoms including having urges to beat, injure, or harm someone, feeling easily annoyed or irritated, or getting into frequent arguments. Across all waves, between 64–76% of respondents reported at least some symptoms of hostility with higher percentages occurring in earlier waves. Symptoms of hostility were consistently reported the highest of these four measures throughout the 10 waves.

#### Independent variables.

*Contact with the criminal legal system.* We use three measures of contact with the criminal legal system measured between the first and last follow-up interviews: being arrested, making a court appearance, and being institutionalized. These binary measures were each coded such that 0 = no and 1 = yes. For the measure of being arrested, respondents were asked if they were arrested in the recall period. Across the waves, between 18 and 29% of the sample reported being arrested within the recall period. For the measure of court appearance, respondents were asked “did you have a court appearance for something you were accused of or up for?” Across waves, between 26 and 43% reported they made a court appearance within the recall period. Finally, respondents reported on their main location during the recall period. The main location included information on whether the respondent was housed in the community (e.g., own house, with family, foster care), or in an institution (e.g., hospital, secure location, prison/jail). We built this measure of institutionalization considering all main criminal legal related institutions including: secure institution, locked facility; jail or prison; detention; and residential treatment center. Between 27 and 48% of respondents in the sample were institutionalized during the recall period across waves.

#### Control variables.

In addition to the relationship between contact with the criminal legal system and mental health outcomes, we controlled for other factors at each wave that have been identified in previous research as relevant and potential confounders of the relationship being explored [16,71,73,84]. Since this is a fixed-effects model examining differences across time within a person, time invariant factors like gender, race, and age at first offense are already accounted for and thus not included in the models. Instead, we included six variables that were dynamic across waves including: being enrolled in school, being employed, number of children, receiving medicine for mental health symptoms, self-reported substance use, and self-reported involvement in illegal activities.

Participants were asked “are you currently enrolled in any type of school?” with 0 = no and 1 = yes. To measure “employment status,” respondents reported if they “had a paying job at any time during the recall period.” Both school enrollment and employment status were about understanding the time use of these respondents. We were interested in what sort of social structures were occupying the time of participants that might serve as a deterrent for delinquent behavior [105] and affect both criminal legal contact and mental health. Respondents were asked to report their number of living children at each wave. We are interested in controlling for number of children [75] as parenthood might affect both contact with the criminal legal system and symptoms of mental health.

Since we are interested in mental health outcomes, we created a variable to delineate if respondents were taking any medication for their mental health during the recall period. The PDS asks respondents to report any medication they are currently taking, and we coded “1” if any of the medication reported could be used for mental health (e.g., antidepressant, antipsychotic, anti-manic) and “0” if all the medication listed were for physical ailments only (e.g., anti-Parkinson, analgesics, anti-convulsant) or if the respondents did not report using any medication at all during the recall period.

We also controlled for respondents’ substance use at each wave. The Substance Use/Abuse Inventory [106] was modified to ask respondents a range of questions about usage of illegal drugs and alcohol during the recall period. The self-report measure is comprised of the following subscales: Substance Use (e.g., “How often have you had alcohol to drink?”) and Social Consequences, Dependency and Treatment (e.g., “Have you ever had problems or arguments with family or friends before because of your alcohol or drug use?”/ “Have you ever wanted a drink or drugs so badly that you could not think about anything else?”). Respondents were coded as “0” if they had no substance use dependency symptoms and “1” if they reported any symptoms of substance use dependency.

Given our interest in contact with the criminal legal system, we control for respondents’ self-reported participation in crime during each recall period. We created a binary variable (none = 0; any reports of offending = 1) from the frequency of offending variable. Some respondents reported hundreds of offenses during the recall period which resulted in an extremely skewed frequency variable, and we decided to focus on the distinction between no offending and any offending.

### Analytic strategy

This study used fixed-effects models to estimate the within-individual changes in mental health symptoms as a result of incremental contacts with the criminal legal system. Fixed-effects modeling was selected as it allows us to assess within-individual changes over time—specifically, whether experiencing different forms of criminal legal contact (i.e., arrest, court appearance, institutionalization) was associated with changes in individual mental health symptoms over a 7-year period. Fixed-effect models control for all stable unobserved variables (e.g., gender, race/ethnicity), while estimating time-varying differences [107–109] allowing us to explore the association between different criminal legal contacts and individual’s mental health symptoms over time.

In this paper we estimated two sets of fixed-effects models. First, we estimated linear fixed-effect models exploring the contribution of arrest, court appearances, and institutionalization to depression, anxiety, hostility, and psychoticism symptoms within the same data collection wave. Second, to expand on this analysis, we estimated additional dynamic fixed-effects models to explore the lagged effects of our three main predictors (measured at t-1) on each of the mental health symptoms. The purpose of these dynamic models is to assess if contacts with the criminal legal system could lead to longitudinal changes in the mental health symptoms experienced by individuals. These secondary analyses models further allow us to explore time ordering effects. In supplementary analyses we re-estimated our two main models, first by adding a lagged version of the dependent variable to ensure the robustness of our findings; and second, exploring how cumulative contact with the criminal legal system was associated with each mental health outcome. This cumulative measure was developed by adding all contacts with the criminal legal system at each wave of data.

We conducted multicollinearity assessments for all analyses and found no issues (VIF ranged between 1.05–1.69 for all variables). Robust standard errors were used in all models due to heteroskedasticity in the data. Despite an 83% retention rate by the final wave of the study there was missingness on the dependent variables (approximately 35% for all variables), thus, we opted to conduct a full case analysis. Supplementary sensitivity analyses (available in S6 Table) were conducted using mean imputation and single-item imputation where missing values were imputed with a 0-no symptoms and 4-extreme symptoms. The findings remained substantially the same for the mean imputation and missing = 0 models. For the missing = 4 models, simulating the most extreme cases possible, all criminal legal contacts predict mental health outcomes, with both courts and institutionalization predicting decreases in mental health symptoms. This may be due to court mandated treatment or access to treatment in institutionalization settings. All analyses were conducted using STATA V.18.

### Ethics statement

The secondary survey data from the Pathways to Desistance Study (PDS) used in this study are available from Inter-university Consortium for Political and Social Research (ICPSR; https://www.icpsr.umich.edu/web/NAHDAP/series/260). As we needed access restricted data from the PDS for this study, we had to follow ICPSR’s restricted data application procedures. First, we had to obtain ethical approval and non-human subjects determination from the Institutional Review Board at Augusta University before requesting access to PDS restricted files. We received ethical approval to conduct the study prior to initiating any research activities (IRB case: 2077730−2, date: 08/17/2023) by the Institutional Review Board at Augusta University. Second, after IRB approval, the application for restricted data access was submitted to ICPSR. After approval and contract signing by all stakeholders, the data was provided by ICPSR in the form of de-identified datasets.

All data used in this project was secondary data collected by the PDS study between November, 2000 – April, 2010. We did not collect any primary data for this study. All consent and assent forms were handled by the original research team.

## Results

### Descriptive statistics

The analytic sample used for this study is comprised of 1,322 individuals. At baseline, this sample is mostly male (84.85%). Most individuals are Black (41%), followed by Hispanic (33%) and White (22%). Throughout all waves of data, the participants’ ages ranged between 14 and 26 years old (*M*_*W1*_ = 16, *M*_*W11*_ = 23). An average of 23% of the sample got arrested at some point after baseline, an average of 33% of participants had a court appearance, and an average of 35% of participants were institutionalized after the baseline assessment. More than half of participants reported mental health symptoms across all waves, with an average of 34% of participants reporting anxiety symptoms after baseline, 41% depression symptoms, 54% hostility symptoms, and 35% reporting psychoticism symptoms after the baseline interview. Descriptive statistics for the sample are presented in Table 1, see also S1 Table in the supplementary materials providing a comparative with the full sample.

**Table 1:** Descriptive statistics for characteristics of the analytical sample.

Variable	Mean	Std.dev.	Min	Max
Arrest	0.233	0.423	0	1
Court appearance	0.329	0.470	0	1
Institutionalization	0.346	0.476	0	1
Residential treatment center	0.068	0.253	0	1
Secure	0.058	0.234	0	1
Jail/Prison	0.209	0.406	0	1
Detention	0.011	0.106	0	1
Cumulative CJ contacts	0.908	0.942	0	3
Anxiety	0.339	0.525	0	4
Depression	0.412	0.622	0	4
Hostility	0.535	0.666	0	4
Psychoticism	0.354	0.546	0	4
In School	0.509	0.500	0	1
Working	0.570	0.495	0	1
Child count	0.425	0.776	0	6
Mental health Medication	0.095	0.294	0	1
Substance use	0.225	0.417	0	1
Participation in crime	0.514	0.500	0	1
Age	19.081	2.345	14	26
Gender (ref. Male)	0.849	0.359	0	1
Race/Ethnicity				
White	0.219	0.414	0	1
Black	0.408	0.492	0	1
Hispanic	0.325	0.468	0	1
Other	0.048	0.213	0	1

*N* = 8,763.

### Linear fixed-effects

Table 2 shows the association between experiencing different contacts with the criminal legal system on anxiety (Model 1), depression (Model 2), hostility (Model 3), and psychoticism (Model 4). Being arrested in the recall period is significantly associated with all four mental health outcomes. Specifically, being arrested is associated with a 0.045 (*p* = 0.006, 95% CI: 0.013–0.077) increase in within-individual reported symptoms of anxiety, an 0.069 (*p* < .001, 95% CI: 0.031–0.108) increase in within-individual reported symptoms of depression, and a 0.036 (*p* = 0.035, 95% CI: 0.002–0.069) increase in within-individual reported symptoms of psychoticism. As for hostility, the association with arrest was also significant, however contrary to all other it was a negative association (*β* = −0.037, *p* = 0.046, 95% CI: −0.075–0.001) indicating that being arrested was associated with a small decrease in the average within-individual hostility symptoms. To put the magnitude of these coefficients into context, the average score for anxiety across all 10 waves was 0.338, for depression 0.412, for hostility 0.535, and for psychoticism 0.354. Based on this, the increase in anxiety, depression, and psychoticism symptoms as a result of arrest are meaningful. Thus, contact with the criminal legal system at the relatively minor level of an arrest, is associated with negative mental health outcomes for anxiety, depression and psychoticism, and a relief in hostility symptoms.

**Table 2:** Results of fixed effects linear regression between criminal legal contacts and mental health symptoms.

	Model 1 Anxiety		Model 2 Depression		Model 3 Hostility		Model 4 Psychoticism	
	Coeff.	RobustS.E.	Coeff.	RobustS.E.	Coeff.	RobustS.E.	Coeff.	RobustS.E.
Arrest	0.045**	0.016	0.069***	0.020	−0.037±	0.020	0.036*	0.017
Court appearances	−0.029*	0.014	0.013	0.017	−0.002	0.018	0.002	0.015
Institutionalization	0.020	0.015	0.126***	0.018	0.071***	0.020	0.078***	0.016
In school	0.018	0.013	0.007	0.015	0.036*	0.016	0.005	0.014
Working	−0.038**	0.013	−0.032*	0.015	−0.036*	0.016	−0.026±	0.013
Child count	0.002	0.012	−0.005	0.013	−0.038**	0.014	−0.023*	0.011
Mental health medication	0.107***	0.030	0.207***	0.035	0.103**	0.036	0.136***	0.032
Substance use	0.084***	0.017	0.143***	0.020	0.111***	0.021	0.128***	0.018
Participating in crime	0.071***	0.012	0.073***	0.014	0.157***	0.017	0.077***	0.013
N	1,322							
N x T	8,763							

*Note*: *p < .05; **p < .01; ***p < .001; ± *p* < .1.

We also explored the relationship between the respondents having a court appearance for something they were accused of and mental health symptoms. Unlike arrest, courts only had a significant short-term association with anxiety. This was a negative relationship indicating that those who had a court appearance had on average a 0.029 decrease in anxiety symptoms (*p* = 0.04, 95% CI: −0,057 – −0.001).

Finally, institutionalization is significantly associated with three of the four outcomes, with increases in depression, hostility, and psychoticism symptoms in the short term. Specifically, institutionalization is associated with an average.126 (*p* < 0.001, 95% CI: 0.0.091–0.161) increase in reported symptoms of depression, a 0.071 (*p* < 0.001, 95% CI: 0.033–0.109) increase in hostility symptoms, and a 0.078 (*p* < 0.001, 95% CI: 0.047–0.109) increase in psychoticism symptoms. There is no significant association between institutionalization and anxiety. Considering the average levels of these symptoms across all waves of data (reported above) these findings are meaningful, especially so for depression symptoms. Based on these findings, institutionalization is associated with increased mental health symptoms of depression, hostility and psychoticism in the short-term, net of other variables.

Regarding control variables, we observed several significant associations with mental health symptoms. Specifically, being employed is associated with a significant reduction in all four mental health outcomes. On the other hand, taking medication for mental health symptoms, substance use, and participating in crime are each associated with increases in levels of anxiety, depression, hostility, and psychoticism.

### Dynamic fixed-effects

Given the potential for time ordering effects, we conducted further analysis using lagged outcomes to examine long-term effects of contact with the criminal legal system on mental health symptoms. To this end, we performed additional analysis estimating dynamic fixed-effects models with lagged measures of the different criminal legal contacts (measured at t – 1). Across these models, both arrest and institutionalization did not have any significant lagged effects on the mental health symptoms measured (Table 3). Court appearances have significant long-term effects on all outcomes. Specifically, appearing in court in one wave significantly increases anxiety symptoms in the following wave by 0.044 (*p* = 0.012, 95% CI: 0.010–0.078), depression by 0.068 (*p* = 0.001, 95% CI: 0.027–0.109), psychoticism by 0.038 (*p* = 0.018, 95% CI: −0.004–0.071), and marginally increases hostility symptoms by 0.033 (*p* = 0.084, 95% CI: 0.003–0.073).

**Table 3:** Results of dynamic fixed effects between criminal legal contacts and mental health symptoms.

	Model 1 Anxiety			Model 2 Depression			Model 3 Hostility			Model 4 Psychoticism		
	Coeff.	Sig.	RobustS.E.	Coeff.	Sig.	RobustS.E.	Coeff.	Sig.	RobustS.E.	Coeff.	Sig.	RobustS.E.
Lagged Arrest	−0.009		0.020	0.003		0.025	0.017		0.025	0.022		0.021
Lagged Court appearances	0.044	*	0.017	0.068	**	0.021	0.033	±	0.019	0.038	*	0.018
Lagged Institutionalization	−0.007		0.018	−0.029		0.021	−0.006		0.022	−0.020		0.019
Time-varying control variables	✓			✓			✓			✓		
N	1,202											
N x T	6,163											

*Note*: *p < .05; **p < .01; ***p < .001; ± *p* < .1.

### Supplementary models

In supplemental analyses we lagged the main outcome variables (anxiety, depression, hostility, and psychoticism) to determine if the presence of prior mental health symptoms predicted the same symptoms at the following wave. The lagged dependent variable was only significant for anxiety (*β* = 0.051, *p* = .042, 95% CI: 0.002–0.100) and depression (*β* = 0.060, *p* = .021, 95% CI: 0.018–0.102). The inclusion of the lagged variable did not substantively change the findings, except for the relationship between arrest and psychoticism which was no longer significant (S1 Table in supplementary files). Similarly, for the dynamic models where the main predictor variables were measured at t-1, the results remained substantively the same (S2 Table in supplementary files).

Finally, we re-estimated the linear and dynamic models exploring the effect of cumulative contacts with the criminal legal system on our four outcome measures. We observe that cumulative contacts with the criminal legal system only have significant short-term effects on depression and psychoticism, increasing these symptoms by 0.055 (*p* < 0.001, 95% CI: 0.039–0.071) and 0.029 (*p* < 0.001, 95% CI: 0.014–0.043) respectively (S4 Table in supplementary files). This said, these cumulative criminal legal contacts have significant long-term effects for depression (*p* = 0.007, 95% CI: 0.008–0.047), hostility (*p* = 0.038, 95% CI: 0.001–0.040), psychoticism *(p* = 0.009, 95% CI: 0.005–0.038), and marginal effects on anxiety (*p* = 0.079, 95% CI: −0.002–0.033). These findings suggest that cumulative contacts with the criminal legal system have more consistent long-term effects on mental health symptoms (S5 Table in supplementary files). Similar to what was observed in the main analyses, taking mental health medication, substance use, and participating in crime were all significantly associated with all mental health outcomes for both linear and dynamic models.

## Discussion

### Discussion of results

Given the overrepresentation of individuals with mental health issues within the criminal legal system, a new line of research exploring the detrimental effects of criminal legal contacts has emerged in recent years. Several studies have proposed that any form of contact with the criminal legal system can be associated with poor health and mental health outcomes in youths and young adults [14,15]. Specifically, studies have suggested that most forms of contact with the criminal legal system are linked to more anxiety and depression symptoms [13,19]. The current study aims at expanding upon this literature and utilizes a high-risk sample of youths and young adults to explore how being arrested, going to court, and being institutionalized are related with short- and long-term mental health symptoms of anxiety, depression, psychoticism and hostility. Four main findings emerged from this study.

First, we observed that arrest may be associated with more short-term symptoms of depression, anxiety, and psychoticism in cross-sectional models. This is in line with prior research which noted that arrests were associated with more depressive symptoms [15]. However, arrest presented a marginally significant but negative association with hostility, suggesting that it reduced short-term hostility symptoms. Further, when we estimated dynamic lagged models, arrest did not have significant effects on any of the outcome variables in the following wave. This seems to suggest that, in our sample, arrest has significant short-term negative effects on mental health symptoms, but does not appear to have long lasting detrimental effects.

Second, similar to arrest, institutionalization was significantly associated with short-term increases in symptoms of depression, psychoticism, and hostility in this study. Further, we observed that institutionalization has no significant lagged effects on any of the measured outcomes in our dynamic models. To the best of our knowledge, no other studies have been conducted using an institutionalization measure, with much of the research being focused exclusively on incarceration. Our findings are counter to what has been found in the incarceration literature, where being incarcerated is reported to have not only immediate but also long-lasting detrimental effects on mental health outcomes and psychological distress [8,12]. We propose that differences in findings in our study may be related to our institutionalization measure. As noted previously, this measure included multiple forms of institutionalization (i.e., residential treatment center, secure institution, jail/prison, detention) and was not limited to only incarceration in a jail or prison. In this way, it is possible that being institutionalized in some capacity, but not necessarily in prison or jail, may have fewer detrimental effects on mental health, and that these may not be as long lasting. Alternatively, in line with prior research [89] institutionalized individuals may have had access to treatment not otherwise available. Finally, these differences in findings may also be due to the age of our respondents. This sample is comprised of youths followed until early adulthood whereas many studies on the detrimental outcomes of incarceration are conducted in adult populations [11,62]. Echoing Baćak and colleagues’ [110] suggestion, there is a need to explore the characteristics of incarceration/institutionalization when exploring their effects on mental health outcomes.

Third, in this study appearing in court did not have any significant cross-sectional effects on most mental health measures with the exception of anxiety where we observe a significant but negative association. This indicates that court appearances seem to decrease short-term anxiety levels. Perhaps this is due to high levels of uncertainty that waiting for a court appearance brings, which are reduced following the court appearance and knowing the outcome. More research is needed to further explore this relationship. In the dynamic models, court appearances had positive significant lagged effects on anxiety, depression, and psychoticism symptoms and a marginal effect on hostility. This suggests that appearing in court may have long-term detrimental effects on psychological well-being. These findings go counter to those by Clemente and Padilla-Racero [93] in their retrospective study of a sample of Spanish adults where they observed immediate negative impacts of attending court not only on anxiety but also hostility, depression, and psychoticism. We attribute these differences at least partially to our longitudinal methodology following a larger sample of youths in the United States; as well as differences in the court systems between countries. While some studies focus on the conviction element of a court appearance [16,17,61,98] more research is needed to explore the relationship between appearing in court for a crime you are accused of and mental health outcomes in longitudinal samples.

Fourth, in our study, the most consistent predictors of mental health outcomes across waves are substance use, participating in crime, and taking mental health medication in both the cross-sectional and lagged models and in all supplementary analyses. These findings are in line with prior research. Specifically, we expected to find a strong association between mental health symptoms and medication to address these symptoms. If individuals are diagnosed with mental health issues frequently, a first line of treatment is to provide medication for symptom management [111]. In line with the literature, there is a strong association, and possible comorbidity, between mental health and substance use [22,43,44]. Finally, some studies have explored the detrimental mental health effects of participating in crime. Research has also suggested that engaging in criminal/illegal activity works as a stressor and increases the presence of mental health symptoms [112,113].

We note that these findings were robust across multiple supplementary analyses in this sample, where arrest and institutionalization were most consistently associated short-term psychological distress and court appearance predicted more long-term psychological distress. Furthermore, we consistently observed in all supplementary models that the most significant and consistent predictors of mental health symptoms were taking mental health medication, substance use, and participating in crime. Finally, we must continue to explore these different forms of contact with the criminal legal system as a continuum, as we found that the cumulative measure of contact with the criminal legal system provided less nuanced information. However, we did find that this cumulative measure was significantly associated with short-term increases in depression and psychoticism symptoms, and long-term increases in all mental health outcomes assessed. These findings align with cumulative disadvantage and life-course research which suggests that contacts with the criminal legal system can work as negative turning points leading individuals to lifelong patterns of continued and accumulated disadvantage in terms of criminal and legal sanctions and health and well-being [84,114–116].

### Implications

This study makes important contributions to criminological theory and has relevant practical health implications. From a criminological theory standpoint, this study expands on Testa and colleagues’ [84] proposal of considering contacts with the criminal legal system as critical life-course events. This approach allows researchers to explore these contacts within a more integrative framework, as opposed to past approaches that almost exclusively considered contacts with the criminal legal system within the deterrence or labelling frameworks. Additionally, we contribute to the knowledge in this area by exploring a continuum of contact with the criminal legal system, including types of contact that have been under-explored in the literature. We also examine other mental health symptoms beyond anxiety and depression. Previous literature [39] has found associations between symptoms of psychoticism and hostility and criminal legal involvement, while failing to explore the possibility that these symptoms could be exacerbated by contact with the criminal legal system.

The current study also has relevant practical implications. In line with previous research on social stress and health [88], our study shows that any contact with the criminal legal system appears to be a stressor that can increase mental health symptoms, having both short-term and long-term effects. This indicates the importance of actively seeking to divert at-risk youth away from contacts with the criminal legal system [117], especially as the size and scope of the US penal system has expanded so dramatically in the past four decades [89]. Educational and community-oriented programs targeting high risk youth, such as after school childcare for elementary age children or vocational training or community centers for teens, could prevent initial involvement in the criminal legal system. This type of programming, especially with an intentional educational focus, would have significant positive implications on the mental health of at-risk individuals. More preventative strategies to reduce offending would allow for less contact with the criminal legal system [118,119] and better mental health.

When youth do end up in the criminal legal system, access to mental health support and services is vital for successful treatment of these symptoms. Importantly, this access should continue after they leave the system to avoid a revolving door issue [48,120–122]. New approaches such as the use of virtual reality in criminal legal settings have been proposed to help reduce the negative effects of contacts with the criminal legal system [123]. This paper highlights the importance of reducing contact with the criminal legal system for mental health outcomes.

### Limitations and future directions

Our study has limitations that should be considered when interpreting results and that could be further explored in future research. First, the sample used in this study includes high risk youths, limiting the generalizability of findings to other low risk samples of youths, or even to different locations from where the data was collected. Future research with lower risk groups, or with comparisons groups including individuals with no criminal legal contact may be relevant. Second, the BSI measure has a limited number of items assessing psychological wellbeing and mental health symptoms over the previous week. Future research should replicate this study using different mental health measures to ensure the generalizability of findings. Third, our measures of contact with the criminal legal system lacked details about these contacts. For instance, we lack information on the reasons for arrest, the outcomes of court proceedings, or specific characteristics of institutions where respondents were held which may have a substantial impact on the mental health symptoms measured. Future research should aim at addressing this limitation by considering not only different forms of contact with the criminal legal system but also details about these contacts. Fourth, while fixed-effects modeling allows us to account for time-invariant characteristics, it is still possible that unobserved variables may impact the outcomes [124]. Fifth, we ultimately did not include a variable related to receiving community services (e.g., a psychologist, counselor, social worker, or mental health treatment group) for mental health symptoms due to high missingness at early waves (up to 33%), but future studies should include measures for non-medicinal support for mental health.

Finally, this study, despite being longitudinal and using lagged models, is still unidirectional, examining the association and effects of contact with the criminal legal system on mental health symptoms. Thus, we did not explore the possibility of reverse causation or reciprocal effects. Based on the literature supporting that contact with the criminal legal system affects mental health, and that those with mental illness are more likely to interact with the criminal legal system, future research should explore reciprocal effects between mental health and criminal legal contact, in line with recent research by Silver and colleagues [20].

## Conclusion

In sum, this study’s key finding is that even less serious forms of contact with the criminal legal system appear to be associated with increases in the number self-reported mental health symptoms in youths and young adults. Specifically, we observed that arrest and institutionalization may have more short-term effects on psychological distress, whereas court appearance appears to have more long-term effects on reported symptoms. These findings suggest that contacts with the criminal legal system are critical life-course events and work as stressors impacting individual mental health, therefore alternative interventions and programming may be effective in reducing mental health issues in these high-risk youths and young adults.

## Supporting information

S1 Table Comparative descriptive statistics for full sample and analytical sample. (DOCX)

S2 Table Results of dynamic fixed effects between criminal legal contacts and lagged mental health symptoms. (DOCX)

S3 Table Results of dynamic fixed effects between criminal legal contacts and mental health symptoms. (DOCX)

S4 Table Results of fixed effects linear regression between cumulative criminal legal contacts and mental health symptoms. (DOCX)

S5 Table Results of dynamic fixed effects between cumulative criminal legal contacts and mental health symptoms. (DOCX)

S6 Table Sensitivity analysis table. (DOCX)
