# Supplementary material for: Effects of differential contacts with the criminal legal system on mental health outcomes of adolescents and young adults: A fixed-effects model
Source: PLoS One. 2026 Jun 17;21(6):e0344895. doi: 10.1371/journal.pone.0344895 (PMC13274883; doi:10.1371/journal.pone.0344895)
Supplement: S1 Table — (DOCX) [file pone.0344895.s001.docx]

**S1 Table**

Comparative descriptive statistics for full sample and analytical sample

|  | **Full**  **Sample** | | **Analytical Sample** | |  |  |
| --- | --- | --- | --- | --- | --- | --- |
| **Variable** | **Mean** | **Std.dev.** | **Mean** | **Std.dev.** | **Min** | **Max** |
| Arrest | 0.221 | 0.415 | 0.233 | 0.423 | 0 | 1 |
| Court Appearance | 0.308 | 0.462 | 0.329 | 0.470 | 0 | 1 |
| Institutionalization | 0.324 | 0.468 | 0.346 | 0.476 | 0 | 1 |
| Residential treatment center | 0.061 | 0.240 | 0.068 | 0.253 | 0 | 1 |
| Secure | 0.051 | 0.219 | 0.058 | 0.234 | 0 | 1 |
| Jail/Prison | 0.202 | 0.401 | 0.209 | 0.406 | 0 | 1 |
| Detention | 0.010 | 0.101 | 0.011 | 0.106 | 0 | 1 |
| Cumulative CJ contacts | 0.853 | 0.937 | 0.908 | 0.942 | 0 | 3 |
| Anxiety | 0.338 | 0.524 | 0.339 | 0.525 | 0 | 4 |
| Depression | 0.412 | 0.622 | 0.412 | 0.622 | 0 | 4 |
| Hostility | 0.354 | 0.546 | 0.535 | 0.666 | 0 | 4 |
| Psychoticism | 0.481 | 0.500 | 0.354 | 0.546 | 0 | 4 |
| In School | 0.575 | 0.494 | 0.509 | 0.500 | 0 | 1 |
| Working | 0.441 | 0.789 | 0.570 | 0.495 | 0 | 1 |
| Child Count | 0.077 | 0.267 | 0.425 | 0.776 | 0 | 6 |
| Mental Health Medication | 0.181 | 0.385 | 0.095 | 0.294 | 0 | 1 |
| Substance Use | 0.449 | 0.497 | 0.225 | 0.417 | 0 | 1 |
| Participation in crime | 0.535 | 0.666 | 0.514 | 0.500 | 0 | 1 |
| Age | 19.215 | 2.353 | 19.081 | 2.345 | 14 | 26 |
| Gender (ref. Male) | 0.841 | 0.343 | 0.849 | 0.359 | 0 | 1 |
| Race/Ethnicity |  |  |  |  |  |  |
| White | 0.202 | 0.402 | 0.219 | 0.414 | 0 | 1 |
| Black | 0.414 | 0.493 | 0.408 | 0.492 | 0 | 1 |
| Hispanic | 0.335 | 0.472 | 0.325 | 0.468 | 0 | 1 |
| Other | 0.048 | 0.214 | 0.048 | 0.213 | 0 | 1 |
