# Supplementary material for: Effects of differential contacts with the criminal legal system on mental health outcomes of adolescents and young adults: A fixed-effects model
Source: PLoS One. 2026 Jun 17;21(6):e0344895. doi: 10.1371/journal.pone.0344895 (PMC13274883; doi:10.1371/journal.pone.0344895)
Supplement: S2 Table — (DOCX) [file pone.0344895.s002.docx]

**S2 Table**

Results of dynamic fixed effects between criminal legal contacts and lagged mental health symptoms

|  | **Model 1**  **Anxiety** | | | **Model 2**  **Depression** | | | **Model 3**  **Hostility** | | | **Model 4**  **Psychoticism** | | |
| --- | --- | --- | --- | --- | --- | --- | --- | --- | --- | --- | --- | --- |
|  | Coeff. |  | Robust S.E. | Coeff. |  | Robust S.E. | Coeff. |  | Robust S.E. | Coeff. |  | Robust S.E. |
| Lagged Dependent Variable | 0.051 | * | 0.025 | 0.060 | ** | 0.021 | 0.030 |  | 0.019 | 0.029 |  | 0.022 |
| Arrest | 0.050 | * | 0.020 | 0.057 | * | 0.026 | -0.057 | * | 0.025 | 0.020 |  | 0.022 |
| Court appearances | -0.040 | * | 0.019 | 0.024 |  | 0.023 | 0.018 |  | 0.024 | 0.003 |  | 0.020 |
| Institutionalization | 0.012 |  | 0.020 | 0.115 | *** | 0.024 | 0.065 | ** | 0.025 | 0.076 | *** | 0.021 |
| In school | 0.020 |  | 0.017 | 0.013 |  | 0.019 | 0.021 |  | 0.020 | -0.006 |  | 0.017 |
| Working | -0.024 |  | 0.016 | -0.015 |  | 0.019 | -0.031 |  | 0.020 | -0.013 |  | 0.018 |
| Child Count | 0.012 |  | 0.015 | 0.005 |  | 0.015 | -0.032 | * | 0.016 | -0.013 |  | 0.014 |
| Mental Health Medicine | 0.126 | ** | 0.038 | 0.222 | *** | 0.043 | 0.103 | * | 0.044 | 0.149 | *** | 0.041 |
| Substance Abuse | 0.087 | *** | 0.021 | 0.137 | *** | 0.024 | 0.095 | *** | 0.025 | 0.117 | *** | 0.022 |
| Criminal Involvement | 0.065 | *** | 0.015 | 0.065 | *** | 0.017 | 0.153 | *** | 0.021 | 0.071 | *** | 0.016 |
| N | 1,202 | | | | | | | | | | | |
| N x T | 6,163 | | | | | | | | | | | |

*Note*: **p* < .05; ***p* < .01; ****p* < .001; ± *p* < .1.

The lagged dependent variable was only significant for anxiety (*p* = .042, 95% CI: 0.002 – 0.100) and depression (*p* = .021, 95% CI: 0.018 – 0.102). When comparing findings from this supplementary analysis integrating the lagged dependent variables at t-1 we find no substantive change in the significance and direction of the findings, with the exception of the relationship between arrest and psychoticism which is no longer significant.
