# Supplementary material for: Effects of differential contacts with the criminal legal system on mental health outcomes of adolescents and young adults: A fixed-effects model
Source: PLoS One. 2026 Jun 17;21(6):e0344895. doi: 10.1371/journal.pone.0344895 (PMC13274883; doi:10.1371/journal.pone.0344895)
Supplement: S3 Table — (DOCX) [file pone.0344895.s003.docx]

**S3 Table**

Results of dynamic fixed effects between criminal legal contacts and mental health symptoms

|  | **Model 1**  **Anxiety** | | | **Model 2**  **Depression** | | | **Model 3**  **Hostility** | | | | **Model 4**  **Psychoticism** | | | |
| --- | --- | --- | --- | --- | --- | --- | --- | --- | --- | --- | --- | --- | --- | --- |
|  | Coeff. | Sig. | Robust S.E. | Coeff. | Sig. | Robust S.E. | Coeff. | Sig. | Robust S.E. | Coeff. | | Sig. | Robust S.E. |  |
| Lagged Dependent Variable | 0.050 | * | 0.025 | 0.066 | ** | 0.021 | 0.032 | ± | 0.019 | 0.031 | |  | 0.022 |  |
| Lagged Arrest | -0.014 |  | 0.020 | -0.005 |  | 0.025 | 0.017 |  | 0.024 | 0.019 | |  | 0.021 |  |
| Lagged Court appearances | 0.045 | ** | 0.017 | 0.067 | ** | 0.021 | 0.033 | ± | 0.019 | 0.038 | | * | 0.018 |  |
| Lagged Institutionalization | -0.009 |  | 0.018 | -0.037 | ± | 0.021 | -0.009 |  | 0.021 | -0.022 | |  | 0.018 |  |
| Time-varying control variables | ✓ | | | ✓ | | | ✓ | | | | ✓ | | | |
| N | 1,202 | | | | | | | | | | | | | |
| N x T | 6,163 | | | | | | | | | | | | | |

*Note*: **p* < .05; ***p* < .01; ****p* < .001; ± *p* < .1

The lagged dependent variable was only significant for anxiety (*p* = .047, 95% CI: 0.001 – 0.066) and depression (*p* = .002, 95% CI: 0.024 – 0.108). When comparing findings from this supplementary analysis integrating the lagged dependent variables a lagged criminal legal contacts at t-1, we find no substantive change in the significance and direction of the findings, with the exception of the marginally significant long-term effect of institutionalization on depression. This suggests that institutionalization is marginally associated with a decrease in the number of depressive symptoms in the following wave.
