# Supplementary material for: Effects of differential contacts with the criminal legal system on mental health outcomes of adolescents and young adults: A fixed-effects model
Source: PLoS One. 2026 Jun 17;21(6):e0344895. doi: 10.1371/journal.pone.0344895 (PMC13274883; doi:10.1371/journal.pone.0344895)
Supplement: S4 Table — (DOCX) [file pone.0344895.s004.docx]

**S4 Table**

Results of fixed effects linear regression between cumulative criminal legal contacts and mental health symptoms

|  | **Model 1**  **Anxiety** | | | **Model 2**  **Depression** | | | **Model 3**  **Hostility** | | | **Model 4**  **Psychoticism** | | |
| --- | --- | --- | --- | --- | --- | --- | --- | --- | --- | --- | --- | --- |
|  | Coeff. |  | Robust S.E. | Coeff. |  | Robust S.E. | Coeff. |  | Robust S.E. | Coeff. |  | Robust S.E. |
| Cumulative CJ Contacts | 0.007 |  | 0.006 | 0.055 | *** | 0.008 | 0.000 |  | 0.009 | 0.029 | *** | 0.008 |
| In school | 0.016 |  | 0.013 | 0.018 |  | 0.015 | 0.053 | ** | 0.016 | 0.013 |  | 0.014 |
| Working | -0.038 | ** | 0.012 | -0.035 | * | 0.014 | -0.040 | * | 0.016 | -0.028 | * | 0.013 |
| Child Count | 0.003 |  | 0.012 | -0.005 |  | 0.013 | -0.039 | ** | 0.014 | -0.023 | * | 0.011 |
| Mental Health Medicine | 0.106 | *** | 0.030 | 0.213 | *** | 0.035 | 0.112 | ** | 0.036 | 0.140 | *** | 0.032 |
| Substance Abuse | 0.084 | *** | 0.017 | 0.131 | *** | 0.020 | 0.096 | *** | 0.021 | 0.120 | *** | 0.018 |
| Criminal Involvement | 0.074 | *** | 0.012 | 0.070 | *** | 0.014 | 0.149 | *** | 0.016 | 0.074 | *** | 0.013 |
| N | 1,322 | | | | | | | | | | | |
| N x T | 8,763 | | | | | | | | | | | |

*Note*: **p* < .05; ***p* < .01; ****p* < .001; ± *p* < .1

When comparing findings from this supplementary analysis integrating the cumulative contacts with the criminal legal system, we observe that cumulative contacts with the criminal legal system only have significant short-term effects on depression (*p* < 0.001, 95% CI: 0.039 - 0.071) and psychoticism (*p* < 0.001, 95% CI 0.014 – 0.043) respectively.
