# Supplementary material for: Effects of differential contacts with the criminal legal system on mental health outcomes of adolescents and young adults: A fixed-effects model
Source: PLoS One. 2026 Jun 17;21(6):e0344895. doi: 10.1371/journal.pone.0344895 (PMC13274883; doi:10.1371/journal.pone.0344895)
Supplement: S5 Table — (DOCX) [file pone.0344895.s005.docx]

**S5 Table**

Results of dynamic fixed effects between cumulative criminal legal contacts and mental health symptoms

|  | **Model 1**  **Anxiety** | | | **Model 2**  **Depression** | | | **Model 3**  **Hostility** | | | **Model 4**  **Psychoticism** | | |
| --- | --- | --- | --- | --- | --- | --- | --- | --- | --- | --- | --- | --- |
|  | Coeff. | Sig. | Robust S.E. | Coeff. | Sig. | Robust S.E. | Coeff. | Sig. | Robust S.E. | Coeff. | Sig. | Robust S.E. |
| Lagged Cumulative CJ Contacts | 0.015 | ± | 0.009 | 0.027 | ** | 0.01 | 0.020 | * | 0.010 | 0.022 | ** | 0.008 |
| Time-varying control variables | ✓ | | | ✓ | | | ✓ | | | ✓ | | |
| N | 1,202 | | | | | | | | | | | |
| N x T | 6,163 | | | | | | | | | | | |

*Note*: **p* < .05; ***p* < .01; ****p* < .001; ± *p* < .1

Cumulative criminal legal contacts appear to have significant long-term effects for depression (*p* = 0.007, 95% CI: 0.008 – 0.047), hostility (*p* = 0.038, 95% CI: 0.001 – 0.040), and psychoticism *(p* = 0.009, 95% CI: 0.005 – 0.038). These findings suggest that cumulative contacts with the criminal legal system have more consistent long-term effects on mental health symptoms than short-term effects (as reported on S3 Table).
