# Supplementary material for: Effects of differential contacts with the criminal legal system on mental health outcomes of adolescents and young adults: A fixed-effects model
Source: PLoS One. 2026 Jun 17;21(6):e0344895. doi: 10.1371/journal.pone.0344895 (PMC13274883; doi:10.1371/journal.pone.0344895)
Supplement: S6 Table — (DOCX) [file pone.0344895.s006.docx]

**S6 Table**

Sensitivity analyses

|  | **Anxiety** | | | **Depression** | | | **Hostility** | | | **Psychoticism** | | |
| --- | --- | --- | --- | --- | --- | --- | --- | --- | --- | --- | --- | --- |
|  | Coeff. |  | Robust S.E. | Coeff. |  | Robust S.E. | Coeff. |  | Robust S.E. | Coeff. |  | Robust S.E. |
| **Minimum case imputation (missing = 0)** | | | | | | | | | | | | |
| Arrest | 0.025 | ± | 0.013 | 0.042 | *** | 0.015 | -0.044 | ** | 0.016 | 0.016 |  | 0.016 |
| Court | -0.018 |  | 0.011 | 0.011 |  | 0.013 | 0.001 |  | 0.014 | 0.005 |  | 0.014 |
| Institutionalization | 0.024 | * | 0.011 | 0.104 | *** | 0.013 | 0.071 | *** | 0.015 | 0.065 | *** | 0.015 |
| Time-varying control variables | ✓ | | | ✓ | | | ✓ | | | ✓ | | |
| **Mean imputation (missing = mean)** | | | | | | | | | | | | |
| Arrest | 0.030 | * | 0.013 | 0.049 | *** | 0.015 | -0.036 | ** | 0.015 | 0.022 | ± | 0.013 |
| Court | -0.025 | * | 0.011 | 0.002 |  | 0.013 | -0.010 |  | 0.014 | -0.002 |  | 0.012 |
| Institutionalization | 0.005 |  | 0.011 | 0.080 | *** | 0.013 | 0.041 | ** | 0.015 | 0.045 | *** | 0.012 |
| Time-varying control variables | ✓ | | | ✓ | | | ✓ | | | ✓ | | |
| **Maximum case imputation (missing = 4)** | | | | | | | | | | | | |
| Arrest | 0.090 | ± | 0.046 | 0.107 | * | 0.046 | 0.107 | * | 0.046 | 0.020 |  | 0.045 |
| Court | -0.102 | ** | 0.039 | -0.073 | ± | 0.039 | -0.073 | ± | 0.039 | -0.084 | * | 0.038 |
| Institutionalization | -0.204 | *** | 0.045 | -0.125 | ** | 0.045 | -0.125 | ** | 0.045 | -0.157 | *** | 0.044 |
| Time-varying control variables | ✓ | | | ✓ | | | ✓ | | | ✓ | | |

*Note*: **p* < .05; ***p* < .01; ****p* < .001; ± *p* < .1

Sensitivity analyses were conducted to determine the potential effects of missing data on our findings. To this end we used mean imputation and single-item imputation where missing values were imputed with a 0-no symptoms (minimum case imputation) and 4-extreme symptoms (maximum case imputation). The findings remained substantially the same for the mean imputation and minimum case imputation (missing=0) models. For the maximum case imputation (missing=4) models, simulating the most extreme cases possible, all criminal legal contacts predict mental health outcomes. Both courts and institutionalization predict decreases in mental health symptoms in this maximum case model. This may be due to court mandated treatment or access to treatment in institutionalization settings.
