# Supplementary material for: Effects of differential contacts with the criminal legal system on mental health outcomes of adolescents and young adults: A fixed-effects model
Source: PLoS One. 2026 Jun 17;21(6):e0344895. doi: 10.1371/journal.pone.0344895 (PMC13274883; doi:10.1371/journal.pone.0344895)
Supplement: Attachment — Submitted filename: PLOS ONE.docx [file pone.0344895.s007.docx]

PLOS ONE

Effects of differential contacts with the criminal justice system on mental health outcomes: A fixed-effects model

This paper’s topic is an important contribution to the literature on youth legal involvement and mental health outcomes. However, framing seems to fit more in line with a criminal justice journal, the focus on youth is often lost, and the methods and presentation of results need particular refinement.

Title and Abstract:

- The paper is focused on youth but the title does not include this
- It would be helpful to include the time periods for follow up.
- The last sentence here should itself be an implication or conclusion rather than a statement that this will be discussed in the manuscript.

Language used:

- I would recommend that the authors use the terminology ‘criminal legal’ rather than ‘criminal justice’ given the lack of justice that the criminal legal system often imposes.
- There is a concerning amount of stigmatizing language used throughout, some examples are highlighted here. Please remove phrases like ‘criminal populations,’ ‘offenders,’ and ‘criminal behaviors’ as this voids the context of what in the United States is criminalized and who is seen as ‘criminal.’ It is also not person-centered language, particularly important in this work. Please say phrases such as ‘criminal legal involved individuals.’ ‘Substance abuse’ is also an outdated term and the scale used includes ‘substance use,’ so I would recommend this language or ‘substance misuse’ when referring to troubling or self-declared troubling substance use.

Introduction:

- Please rephrase that ‘research has suggested the opposite’ as both bodies of research say that heightened mental health needs and heightened criminal involvement move in the same direction. It is a question of direction of causality. However, the authors are not exploring direction of causality, so this introduction is confusing.
- Please expand on what the ‘continuum of contact’ entails from prior work.
- Define ‘institutionalization’ the first time it is used, as this often refers to institutions outside of criminal legal settings such as mental health hospitals. While ‘including facilities designed for minors’ is more specific, it is still unclear if this is state prison system facilities for youth, county-level facilities for youth, or other others.
- Clarify the following: (1) intermediate contact vs. lower-level contact like probation and (2) going to trial seems to be muddled with pre-trial jail time.
- Change ‘higher mental health issues’ to ‘higher prevalence of mental health issues.’
- I would recommend highlighting the high prevalence of legal-involved youth compared to legal-involved adults as well.
- The statement that only a small proportion of crimes are directly linked to mental illness seems too narrow. Given the co-occurrence of mental illness and substance use – often a coping strategy or self-medicating strategy – many ‘crimes’ are very closely tied to one’s mental illness. The three primary pathways described almost further this issue because they all related to substance use.
- The statement on a need for treatment programs seems out of place.
- Given the focus of the manuscript, I would recommend a criminology-focused journal. The focus on desistance, delinquency, and individual-level behavior rather than socioecological or structural factors that shape individuals’ trajectories is more aligned with criminology. If keeping here, I would recommend including the stress process model or other more structural models.
- Primary stressor isn’t necessary ‘brief and limited’ if that one sentence is quite long.
- ‘Continuum of contact’ section can easily fit with the above section and citing similar studies. For example, it is odd that Sugie and Turney’s piece isn’t cited until the later section.
- I question the focus on adult versus adolescent studies given that the focus is on adolescents. I understanding nodding to and including adult studies but it removes the context away from youth by focusing on them without the caveat throughout of how this may look different from youth.

Methods:

- Define ‘adjudicated’
- ‘Good representation of female offenders’ – please expand on this by stating the percent of female youth involved in the system versus those included in the study.
- State retention percentage across waves and how those without complete data were treated. Later, it says that only those with the outcome variable were included, but it is not clear more broadly over time.
- Move all descriptive statistics to the results section. This will also shorten the ‘control variables’ section and the individual paragraphs for each control variable can be removed. Descriptive statistics on mental health medication should be reported in conjunction with the % who qualify for mental health medication.
- If using ‘institutionalization’ then need to remove ‘incarcerated’ as the label here, as this includes secure institutions, locked facilities, and residential treatment centers. Please also present descriptive statistics (in the results) for the various forms of institutions. It is stated in the limitations that this is not available, but please say that upfront if it is not possible at all to disentangle.
- The control variables require a more thorough justification. Are they confounding variables and thus impact exposure and outcome?
  - Education: is there a way to separate out high school or GED from higher education, as this seems quite distinct?
  - If the individual does not have a paying job, that does not necessarily make them unemployed, as they need to be wanting and seeking work – particularly while they are still minors.
- The models allow you to assess relationships between exposure and outcome, not control variables and outcome. I am concerned by the language ‘we were interested in what sorts of social structures…’ because that implies that you are interpreting associations from control variables.
- A table comparing the final versus full sample and p-values for important differences would be helpful.
- Sensitivity analyses and a brief statement of similarity or difference of findings should be included as a supplement.

Results

- Please present confidence intervals, as they show both precision and accuracy of estimates.
- The findings are stated as ‘meaningful’ but lacking context to support this.
- Remove paragraph on associations from control variables, as the model is meant to control for the exposure-outcome relationship, not control-outcome relationship.
- I would recommend presenting the dynamic model alongside the initial model for each exposure to directly compare. Or, selecting one as the primary method and the other as a secondary analysis. It’s currently hard to follow from both. The dynamic model description also reads similarly to the first supplementary model.
- Move discussion of imputation to the methods.

Discussion:

- Emphasize focus on youth, which is one of the primary contributions.
- I would reduce the restating of results in the discussion and tone down the finality of the findings. For example, arrests may have other longer-term impacts on mental health, but other work is not cited here.
- I caution again about the comparison to other incarceration work when this outcome combines incarceration with other forms of institutionalization.
- No citations are provided for the potential benefits of incarceration.
- Remove interpretation of control variables here as well.

Implications

- The relevance to criminological theory should be moved to the discussion. However, this should also be expanded on to include public health literature on the topic that focuses less on criminological factors and more on structural and system factors.
- The practical implications should also be moved to the discussion.
- Then, the implications paragraph should more summarize the main findings, future directions, and brief implications.
