# Supplementary material for: Effects of differential contacts with the criminal legal system on mental health outcomes of adolescents and young adults: A fixed-effects model
Source: PLoS One. 2026 Jun 17;21(6):e0344895. doi: 10.1371/journal.pone.0344895 (PMC13274883; doi:10.1371/journal.pone.0344895)
Supplement: Attachment — Submitted filename: Response to reviewers.docx [file pone.0344895.s009.docx]

Dear Dr. Knittel,

My co-author and I were pleased to receive your response inviting us to revise and resubmit our manuscript. Accordingly, we would like to submit the enclosed revised paper, “Effects of differential contacts with the criminal legal system on mental health outcomes of adolescents and young adults: A fixed-effects model,” Manuscript #PONE-D-25-43024, for reconsideration for publication in PLOS ONE.

We thank you and the reviewers for the time and effort in reviewing our manuscript. The feedback has been invaluable in improving the content and presentation of the paper. We have revised the manuscript according to the reviewers’ comments, and our point-by-point responses are given below (page and line numbers based on the clean manuscript). Attached are the manuscripts with track changes and a clean manuscript as requested.

**Answer to comments from Editor:**

1. Please ensure that your manuscript meets PLOS ONE's style requirements, including those for file naming. The PLOS ONE style templates can be found at

https://journals.plos.org/plosone/s/file?id=wjVg/PLOSOne_formatting_sample_main_body.pdf and

https://journals.plos.org/plosone/s/file?id=ba62/PLOSOne_formatting_sample_title_authors_affiliations.pdf

**Answer: Edits were made according to the instructions provided.**

2. Please include your full ethics statement in the ‘Methods’ section of your manuscript file. In your statement, please include the full name of the IRB or ethics committee who approved or waived your study, as well as whether or not you obtained informed written or verbal consent. If consent was waived for your study, please include this information in your statement as well.

**Answer: The ethics statement can be found on page 20, line 409 and includes all the required elements.**

3. We note you have included a table to which you do not refer in the text of your manuscript. Please ensure that you refer to Table 3 in your text; if accepted, production will need this reference to link the reader to the Table.

**Answer: We made the edits and added this information to page 26, line 487.**

4. Please include captions for your Supporting Information files at the end of your manuscript, and update any in-text citations to match accordingly. Please see our Supporting Information guidelines for more information: <http://journals.plos.org/plosone/s/supporting-information>.

**Answer: We added a Supporting Information Captions section at the end of the manuscript (page 44, line 1031) and supplemental files were edited to comply with the journal’s requirements.**

**Answer to comments from Reviewer 1:**

1. This was a very extensive introduction. While PLOSOne does not offer a specific word count limit, much of the background information in the introduction could be presented more concisely.

**Answer: We summarized and reduced the background section as much as possible while still making the other requested edits to the manuscript.**

2. Lines 90-92 “Only a very small…especially psychotic symptoms.”: As written, it is unclear whether “especially psychotic symptoms” means psychotic symptoms are more or less likely to be linked to crimes That sentence could be saying that (1) the small proportion of crimes that are linked to mental illness symptoms are usually specifically linked to psychotic symptoms, or (2) that within the small proportion of crimes linked to mental illness symptoms, an even smaller portion of crimes are linked to psychotic symptoms. Please rephrase to clarify.

**Answer: To clarify this point, the sentence was removed and the paragraph edited on page 4, lines 82-88.**

3. In the paragraph from lines 93-99, you introduce the 3 possible distinct pathways connecting mental health and criminal legal involvement but then the following 3 paragraphs where the authors describe each in detail are not really aligned with how they are initially introduced.

**Answer: We reworked this section so that the introductory paragraph and description of the pathways match, now beginning on page 5, line 89.**

4. The relationship between criminal justice contact and mental illness are complex. There is the pathway that both criminal legal contact and mental illness have common risk factors. For example, childhood trauma increases risk of both mental illness and criminal legal involvement. While I believe this might be addressed in the first pathway referring to mediating or moderating factors (“other social factors”), it is important to make this clearer if so.

**Answer: This point has been clarified on page 5 beginning on line102, within the rewrite for the different pathways and in the description of control variables selected for this study beginning on page 16 line 331.**

5. Also, this may just be disciplinary differences or preference but from my perspective as a clinician-scientist, mental illness includes substance use disorders and personality disorders, so when SUD and personality disorders are described as mediating/moderating factors between mental illness and crime – it is confusing. This just may be a difference between health sciences and criminology but it may be helpful to make it clear that this study considers SUD and mental illness separately.

**Answer: We added a clarification of how the different types of psychiatric diagnosis are classified in our study and cited prior literature using the same/similar classifications. These edits can now be found on page 5 lines 97-102.**

6. Use “substance use disorder” consistently throughout. There are some instances where the term “substance abuse disorders” is used.

**Answer: This change was made throughout the paper, and we now only use the term “substance use” (or substance use disorder, where appropriate).**

7. Lines 110-116: This paragraph does not really describe what that authors are referring to a “reverse causation,” it instead just reiterates the prevalence of substance use disorders in justice involved populations. I would have expected evidence for describing this pathway to include various ways in which incarceration and broader criminal legal involvement may harm mental health including studies about how solitary confinement, lengths of incarceration, violence and trauma exposures during incarceration, arrests and negative police encounters have all been associated with depression, PTSD, and other mental health symptoms.

**Answer: This paragraph has been rewritten to better reflect the reverse causation element. It can now be found on page 6 lines 115-122.**

8. Lines 117-125: This paragraph refers to the reciprocal/bidirectional nature of the mental health and criminal legal involvement, which encompasses what I would have expected in the prior paragraph about “reverse causation.” Overall, the description of and distinction between the 3 pathways should be clearer

**Answer: Based on comments and suggestions made by the reviewer, this section of the paper was fully reworked to make these distinctions clear. The rewritten pathways section begins on page 5 and continues through to page 6.**

**Answer to comments from Reviewer 2:**

*Title and Abstract:*

1. The paper is focused on youth but the title does not include this

**Answer: We have now included that information in the revised title: “Effects of differential contacts with the criminal legal system on mental health outcomes of adolescents and young adults: A fixed-effects model.”**

2. It would be helpful to include the time periods for follow up.

**Answer: We have now added the data collection period in the abstract briefly and this information is expanded upon in more detail in the “Data and participants” part of the Methods section (page 13, lines 267-269).**

3. The last sentence here should itself be an implication or conclusion rather than a statement that this will be discussed in the manuscript.

**Answer: We have now added an additional sentence to the end of the abstract, summarizing our conclusions. “Thus, our paper evidences that even less severe contacts with the criminal legal system appear to be stressors that can have both short-term and long-term effects on mental health symptoms in youths and young adults.” (page 2, lines 34-36)**

*Language used:*

4. I would recommend that the authors use the terminology ‘criminal legal’ rather than ‘criminal justice’ given the lack of justice that the criminal legal system often imposes.

**Answer: We have gone through and made changes in this language throughout the paper.**

5. There is a concerning amount of stigmatizing language used throughout, some examples are highlighted here. Please remove phrases like ‘criminal populations,’ ‘offenders,’ and ‘criminal behaviors’ as this voids the context of what in the United States is criminalized and who is seen as ‘criminal.’ It is also not person-centered language, particularly important in this work. Please say phrases such as ‘criminal legal involved individuals.’ ‘Substance abuse’ is also an outdated term and the scale used includes ‘substance use,’ so I would recommend this language or ‘substance misuse’ when referring to troubling or self-declared troubling substance use.

**Answer: We have gone through and made changes to this language throughout paper – we no longer use any of the terms mentioned and consistently use the term substance use in the paper.**

*Introduction:*

6. Please rephrase that ‘research has suggested the opposite’ as both bodies of research say that heightened mental health needs and heightened criminal involvement move in the same direction. It is a question of direction of causality. However, the authors are not exploring direction of causality, so this introduction is confusing.

**Answer: The sentence has been rephrased based on this comment. It is relevant to clarify** **we are assessing causal/time ordering (not causality) in the current paper by using fixed effects modeling and lagged models (with covariates in the same wave and mental health outcomes measured at later waves).**

7. Please expand on what the ‘continuum of contact’ entails from prior work.

**Answer: We have now added more details about what we mean by “continuum of contact” in the Introduction (page 3, lines 54-57) better connecting it to the section on “continuum of contact” where describe prior research on this topic (page 11, line 222),**

8. Define ‘institutionalization’ the first time it is used, as this often refers to institutions outside of criminal legal settings such as mental health hospitals. While ‘including facilities designed for minors’ is more specific, it is still unclear if this is state prison system facilities for youth, county-level facilities for youth, or other others.

**Answer: We have now added a definition of “institutionalization” as we operationalize it in our study the first time that we mention the term in the manuscript on page 3 lines 56-57.**

9. Clarify the following: (1) intermediate contact vs. lower-level contact like probation and (2) going to trial seems to be muddled with pre-trial jail time.

**Answer: This wording has been removed/corrected throughout the paper to make our concepts less subjective and clearer.**

10. Change ‘higher mental health issues’ to ‘higher prevalence of mental health issues.’

**Answer: We have now corrected this typo on page 4 line 77.**

11. I would recommend highlighting the high prevalence of legal-involved youth compared to legal-involved adults as well.

**Answer: We would appreciate clarification on this point so that we can make the suggested edit. We do present percentages of mental health diagnoses for both youths and adults in the criminal legal system as compared to the general population under the section “Mental health and the criminal legal system” on pages 3-4.**

12. The statement that only a small proportion of crimes are directly linked to mental illness seems too narrow. Given the co-occurrence of mental illness and substance use – often a coping strategy or self-medicating strategy – many ‘crimes’ are very closely tied to one’s mental illness. The three primary pathways described almost further this issue because they all related to substance use.

**Answer: We have now rewritten this section on the three primary pathways, now beginning on page 5, line 89.**

13. The statement on a need for treatment programs seems out of place.

**Answer: We removed this out of place statement from the manuscript.**

14. Given the focus of the manuscript, I would recommend a criminology-focused journal. The focus on desistance, delinquency, and individual-level behavior rather than socioecological or structural factors that shape individuals’ trajectories is more aligned with criminology. If keeping here, I would recommend including the stress process model or other more structural models.

**Answer: We have now added a paragraph incorporating social stress and the Stress Process Model on pages 7-8. We agree this project ties in well to this literature.**

15. Primary stressor isn’t necessary ‘brief and limited’ if that one sentence is quite long.

**Answer: The language was edited based on this suggestion. Changes can now be seen on page 10, line 213.**

16. ‘Continuum of contact’ section can easily fit with the above section and citing similar studies. For example, it is odd that Sugie and Turney’s piece isn’t cited until the later section.

**Answer: We opted to keep the continuum of contact as its own separate section due to its relevance to our own paper and data analysis. That said, we did make edits to clarify links between this section and the previous section that will hopefully help the reader understand why we are presenting the narrative in this manner. Please note that we consider the Sugie and Turney piece extremely important literature and we cite it in two places before this citation (on pages 5 and 10).**

17. I question the focus on adult versus adolescent studies given that the focus is on adolescents. I understanding nodding to and including adult studies but it removes the context away from youth by focusing on them without the caveat throughout of how this may look different from youth.

**Answer: As our sample follows individuals from age 14 at wave 1 to age 25 at wave 11, we find the need to include both literature on teenagers and adults in this paper.**

*Methods:*

18. Define ‘adjudicated’

**Answer: We have added a definition added on page 12 line 256.**

19. ‘Good representation of female offenders’ – please expand on this by stating the percent of female youth involved in the system versus those included in the study. –

**Answer: We rephrased this section to more closely match what the authors reported in their seminal piece. In the original paper, the authors state “(c) a sizable enough number of female serious offenders to examine sex differences in patterns of development and desistance from crime;” (Schubert et al, 2004, p.242). Unfortunately, we do not have access to information about the number of female youth in the system during the time period and locations where the data were collected so we cannot make this comparison.**

20. State retention percentage across waves and how those without complete data were treated. Later, it says that only those with the outcome variable were included, but it is not clear more broadly over time.

**Answer: The retention rate for the whole study was 83%, in the final wave there were 1,134 participants who completed all 10 follow-up interviews. This information was added to page 13 lines 274-277. This was further clarified beginning on page 20, line 424.**

21. Move all descriptive statistics to the results section. This will also shorten the ‘control variables’ section and the individual paragraphs for each control variable can be removed. Descriptive statistics on mental health medication should be reported in conjunction with the % who qualify for mental health medication.

**Answer: We moved the descriptive statistics to the results section as requested. This section now begins on page 20, line 423. Unfortunately, information on individuals who *qualify* to receive medication is not available in this dataset, only information on who reports currently taking a medication for their mental health (e.g., antidepressant, antipsychotic, anti-manic). That information is introduced on page 17 and then reported in Table 1 on page 22.**

22. If using ‘institutionalization’ then need to remove ‘incarcerated’ as the label here, as this includes secure institutions, locked facilities, and residential treatment centers. Please also present descriptive statistics (in the results) for the various forms of institutions

**Answer: We corrected the wording based on this suggestion. We added the descriptive statistics about the types of institution tables both in the manuscript and in Table 1 (page 437) which has been expanded to include types of facilities. We also include this information in Supplemental Table 1 showing the full sample and the analytical sample (S1 Table).**

23. The control variables require a more thorough justification. Are they confounding variables and thus impact exposure and outcome?

**Answer: We have more clearly explained the reasoning for our control variables beginning on page 16 with citations linking why these factors are relevant to include in this study.**

23.1. Education: is there a way to separate out high school or GED from higher education, as this seems quite distinct?

**Answer: Unfortunately, we don’t have information about high school versus GED or about specific educational levels. However, for this study, we are focused on the importance of being currently enrolled in school (or not) which is seen as important across several criminological theories (e.g., social bonds, age graded theory of social control, Moffit’s taxonomy). We note that *not* being in school is a strong risk factor for criminal involvement due to a number of factors, including a lack of social controls that come from being in school all day. We now specify this on pages 16-17 of the manuscript (lines 342-345) including the relevant citations.**

23.2. If the individual does not have a paying job, that does not necessarily make them unemployed, as they need to be wanting and seeking work – particularly while they are still minors.

**Answer: To measure “employment status,” respondents were asked to report if they “had a paying job at any time during the recall period.” Unfortunately, there was no information in the survey about their reasons for unemployment or if they were currently seeking a job. However, similar to being enrolled in school, we view the importance of this variable as being a measure of if their time is occupied, reducing their risk of criminal involvement and increasing stakes in conformity (e.g., social bonds theory, age graded theory of social control). We now specify this in the manuscript on pages 16-17 (lines 341-345).**

24. The models allow you to assess relationships between exposure and outcome, not control variables and outcome. I am concerned by the language ‘we were interested in what sorts of social structures…’ because that implies that you are interpreting associations from control variables.

**Answer: In the social sciences, it is common to interpret relationships of control variables as they are related to the dependent variable in fixed-effects models (e.g., Allison, 2009). This approach is also utilized in other articles published with PLOS One using fixed effects modeling (e.g. Larsen et al, 2023, Van Parys, 2016). If there is a need to remove these interpretations, we would appreciate further clarification from the reviewer.**

References:

**Allison, P. D. (2009). *Fixed effects regression models*. SAGE publications.**

**Larsen, F. B., Lasgaard, M., Willert, M. V., & Sørensen, J. B. (2023). Estimating the causal effects of work-related and non-work-related stressors on perceived stress level: A fixed effects approach using population-based panel data. *Plos one*, *18*(8), e0290410.**

**Van Parys, J. (2016). Variation in physician practice styles within and across emergency departments. *PloS one*, *11*(8), e0159882.**

25. A table comparing the final versus full sample and p-values for important differences would be helpful.

**Answer: We have now included a supplemental table with descriptive statistics for both the full sample and the analytical sample (See Supplemental Table 1 (S1): Comparative descriptive statistics for full sample and analytical sample.) It was not possible to present p-values for differences between the two groups as one of these is a subsample of the other, thus violating the independence assumptions of statistical tests that traditionally allow to compare two independent groups.**

26. Sensitivity analyses and a brief statement of similarity or difference of findings should be included as a supplement.

**Answer: We have included the sensitivity analyses and the requested statement as Supplementary Table 5 (S5 Table: Results of dynamic fixed effects between cumulative criminal legal contacts and mental health symptoms).**

*Results*

27. Please present confidence intervals, as they show both precision and accuracy of estimates.

**Answer: We have added confidence intervals to the text as requested. These are throughout the text, beginning on page 23 of the Results section and continuing until just before the Discussion on page 29. These were also added to supplemental files.**

28. The findings are stated as ‘meaningful’ but lacking context to support this.

**Answer: We clarified the context in which these findings are meaningful by referring to the average values of our variables. Edits can be found on page 23, line 455.**

29. Remove paragraph on associations from control variables, as the model is meant to control for the exposure-outcome relationship, not control-outcome relationship.

**Answer: As noted in the response to point 24 under the Methods section, it is common to interpret the covariates in social sciences, and such an approach has been used in other papers in this journal. We would appreciate further clarification before removing these interpretations.**

30. I would recommend presenting the dynamic model alongside the initial model for each exposure to directly compare. Or, selecting one as the primary method and the other as a secondary analysis. It’s currently hard to follow from both. The dynamic model description also reads similarly to the first supplementary model.

**Answer: Based on this comment, we have now clarified that the dynamic models are additional analyses both in the methods section (page 19, line 385) and results (page 26, line 482). The models are similar, but in the dynamic models the main predictors are measured at t-1 allowing us to estimate longitudinal effects and time ordering.**

31. Move discussion of imputation to the methods.

**Answer: We moved this paragraph on imputation to the Analytic Strategy section on page 19 lines 400-408.**

*Discussion:*

32. Emphasize focus on youth, which is one of the primary contributions.

**Answer: Previous studies such as Sugie and Turney (2017), Fernandes (2020), and Boen (2020) have also focused on youth samples while examining differential contacts on health outcomes. We agree there is a need to highlight the main contributions of our study more clearly. Regarding our sample we did clarify that we have a sample of *high-risk* youths and young adults with a criminal record and formal adjudication as, opposed to general population cohorts that include some individuals who participate in illegal activities used in prior research (page 29, line 529). Based on this comment, we also highlighted that our study’s unique contributions, including the high-risk sample, as well as the addition of the mental health outcomes of psychoticism and hostility, and our particular measurements for contact with the criminal legal system, including our measure of institutionalization.**

33. I would reduce the restating of results in the discussion and tone down the finality of the findings. For example, arrests may have other longer-term impacts on mental health, but other work is not cited here.

**Answer: We reorganized the discussion in several ways which led to a discussion of results section that is more reduced and summarized, and to a change in wording to tone down the finality of findings as requested. Please note that the papers cited in the discussion were the ones we could find on the arrest measure, to the best of our knowledge most of the research is on police stops and not arrests.**

34. I caution again about the comparison to other incarceration work when this outcome combines incarceration with other forms of institutionalization.

**Answer: We made adjustments to this section of the discussion to caution the reader regarding comparisons between our institutionalization measure and prior incarceration research. We still established parallels to this literature, as our institutionalization measure does include incarceration, but based on this feedback we made it very clear that there are differences in measurement that need to be considered.**

35. No citations are provided for the potential benefits of incarceration.

**Answer: This section was rewritten to clarify the point made, and we kept the Massoglia and Remster (2019) citation to support that argument.**

36. Remove interpretation of control variables here as well.

**Answer: As noted in the previous sections, the interpretation of control variables is recurrent in the social sciences and provides additional information about outcomes of interest. We would appreciate further clarification before removing these interpretations.**

*Implications*

37. The relevance to criminological theory should be moved to the discussion. However, this should also be expanded on to include public health literature on the topic that focuses less on criminological factors and more on structural and system factors.

**Answer: Although the Implications section was a subsection of the Discussion, based on the reviewer’s feedback we moved this section up (now located before the Limitations section on page 33 beginning on line 609) and rewrote portions of it to better incorporate** **links to health and the stress process model (as previously requested).**

38. The practical implications should also be moved to the discussion.

**Answer: We addressed this by placing the Implications section immediately following the Discussion of the Results (but still in the Discussion section). Practical implications begin on page 33 line 623.**

39. Then, the implications paragraph should more summarize the main findings, future directions, and brief implications.

**Answer: We begin our Discussion section by summarizing the main findings, then move on to implications, then limitations and future directions, and finally we have added a conclusion paragraph to address these suggestions.**

We thank the Editor and the Reviewers for their feedback and hope we have answered your suggestions to your satisfaction.

Sincerely,

The authors
