# Supplementary material for: Effects of differential contacts with the criminal legal system on mental health outcomes of adolescents and young adults: A fixed-effects model
Source: PLoS One. 2026 Jun 17;21(6):e0344895. doi: 10.1371/journal.pone.0344895 (PMC13274883; doi:10.1371/journal.pone.0344895)
Supplement: Peer review history

# Peer review: Decision Letter 0

**DOI:** 10.1371/journal.pone.0344895.r001

**Authors:** Andrea K. Knittel

27 Oct 2025

PONE-D-25-43024

Effects of differential contacts with the criminal justice system on mental health outcomes: A fixed-effects model

PLOS ONE

Dear Dr. V. Oliveira,

Thank you for submitting your manuscript to PLOS ONE. After careful consideration, we feel that it has merit but does not fully meet PLOS ONE’s publication criteria as it currently stands. Therefore, we invite you to submit a revised version of the manuscript that addresses the points raised during the review process.

Specifically, the reviewers have identified issues with the extent and clarity of the methods (Criterion 3) and that the conclusions are clearly linked with the data and the findings (Criterion 4). Please attend to each of the recommendations of the reviewers as you revise your manuscript.

Please submit your revised manuscript by Dec 11 2025 11:59PM. If you will need more time than this to complete your revisions, please reply to this message or contact the journal office at plosone@plos.org. When you're ready to submit your revision, log on to https://www.editorialmanager.com/pone/ and select the 'Submissions Needing Revision' folder to locate your manuscript file.

Please include the following items when submitting your revised manuscript:

- A rebuttal letter that responds to each point raised by the academic editor and reviewer(s). You should upload this letter as a separate file labeled 'Response to Reviewers'.
- A marked-up copy of your manuscript that highlights changes made to the original version. You should upload this as a separate file labeled 'Revised Manuscript with Track Changes'.
- An unmarked version of your revised paper without tracked changes. You should upload this as a separate file labeled 'Manuscript'.

If you would like to make changes to your financial disclosure, please include your updated statement in your cover letter. Guidelines for resubmitting your figure files are available below the reviewer comments at the end of this letter.

If applicable, we recommend that you deposit your laboratory protocols in protocols.io to enhance the reproducibility of your results. Protocols.io assigns your protocol its own identifier (DOI) so that it can be cited independently in the future. For instructions see: https://journals.plos.org/plosone/s/submission-guidelines#loc-laboratory-protocols. Additionally, PLOS ONE offers an option for publishing peer-reviewed Lab Protocol articles, which describe protocols hosted on protocols.io. Read more information on sharing protocols at https://plos.org/protocols?utm_medium=editorial-email&utm_source=authorletters&utm_campaign=protocols.

We look forward to receiving your revised manuscript.

Kind regards,

Andrea K. Knittel

Academic Editor

PLOS ONE

Journal Requirements:

When submitting your revision, we need you to address these additional requirements.

1. Please ensure that your manuscript meets PLOS ONE's style requirements, including those for file naming. The PLOS ONE style templates can be found at

https://journals.plos.org/plosone/s/file?id=wjVg/PLOSOne_formatting_sample_main_body.pdf and

https://journals.plos.org/plosone/s/file?id=ba62/PLOSOne_formatting_sample_title_authors_affiliations.pdf

2. Please include your full ethics statement in the ‘Methods’ section of your manuscript file. In your statement, please include the full name of the IRB or ethics committee who approved or waived your study, as well as whether or not you obtained informed written or verbal consent. If consent was waived for your study, please include this information in your statement as well.

3. We note you have included a table to which you do not refer in the text of your manuscript. Please ensure that you refer to Table 3 in your text; if accepted, production will need this reference to link the reader to the Table.

4. Please include captions for your Supporting Information files at the end of your manuscript, and update any in-text citations to match accordingly. Please see our Supporting Information guidelines for more information: http://journals.plos.org/plosone/s/supporting-information.

5. If the reviewer comments include a recommendation to cite specific previously published works, please review and evaluate these publications to determine whether they are relevant and should be cited. There is no requirement to cite these works unless the editor has indicated otherwise.

[Note: HTML markup is below. Please do not edit.]

Reviewers' comments:

Reviewer's Responses to Questions

**Comments to the Author**

1. Is the manuscript technically sound, and do the data support the conclusions?

The manuscript must describe a technically sound piece of scientific research with data that supports the conclusions. Experiments must have been conducted rigorously, with appropriate controls, replication, and sample sizes. The conclusions must be drawn appropriately based on the data presented.

Reviewer #1: Yes

Reviewer #2: Partly

**********

2. Has the statistical analysis been performed appropriately and rigorously?

Reviewer #1: I Don't Know

Reviewer #2: I Don't Know

**********

3. Have the authors made all data underlying the findings in their manuscript fully available?

The PLOS Data policy requires authors to make all data underlying the findings described in their manuscript fully available without restriction, with rare exception (please refer to the Data Availability Statement in the manuscript PDF file). The data should be provided as part of the manuscript or its supporting information, or deposited to a public repository. For example, in addition to summary statistics, the data points behind means, medians and variance measures should be available. If there are restrictions on publicly sharing data—e.g. participant privacy or use of data from a third party—those must be specified.

Reviewer #1: Yes

Reviewer #2: No

**********

4. Is the manuscript presented in an intelligible fashion and written in standard English?

PLOS ONE does not copyedit accepted manuscripts, so the language in submitted articles must be clear, correct, and unambiguous. Any typographical or grammatical errors should be corrected at revision, so please note any specific errors here.

Reviewer #1: Yes

Reviewer #2: Yes

**********

5. Review Comments to the Author

Please use the space provided to explain your answers to the questions above. You may also include additional comments for the author, including concerns about dual publication, research ethics, or publication ethics. (Please upload your review as an attachment if it exceeds 20,000 characters)

Reviewer #1: Overall summary:

This is a very interesting and insightful study examining the mental health effects of differential contacts with the criminal legal system on mental health. The authors effectively used a longitudinal dataset comprising of youth deems at risk to answer this research question and provide a substantial contribution to academic literature in this area. Most of my concerns are in regard to the intro/background sections.

Introduction

• This was a very extensive introduction. While PLOSOne does not offer a specific word count limit, much of the background information in the introduction could be presented more concisely.

• Lines 90-92 “Only a very small…especially psychotic symptoms.”: As written, it is unclear whether “especially psychotic symptoms” means psychotic symptoms are more or less likely to be linked to crimes. That sentence could be saying that (1) the small proportion of crimes that are linked to mental illness symptoms are usually specifically linked to psychotic symptoms, or (2) that within the small proportion of crimes linked to mental illness symptoms, an even smaller portion of crimes are linked to psychotic symptoms. Please rephrase to clarify.

• In the paragraph from lines 93-99, you introduce the 3 possible distinct pathways connecting mental health and criminal legal involvement but then the following 3 paragraphs where the authors describe each in detail are not really aligned with how they are initially introduced.

• The relationship between criminal justice contact and mental illness are complex. There is the pathway that both criminal legal contact and mental illness have common risk factors. For example, childhood trauma increases risk of both mental illness and criminal legal involvement. While I believe this might be addressed in the first pathway referring to mediating or moderating factors (“other social factors”), it is important to make this clearer if so.

• Also, this may just be disciplinary differences or preference but from my perspective as a clinician-scientist, mental illness includes substance use disorders and personality disorders, so when SUD and personality disorders are described as mediating/moderating factors between mental illness and crime – it is confusing. This just may be a difference between health sciences and criminology but it may be helpful to make it clear that this study considers SUD and mental illness separately.

• Use “substance use disorder” consistently throughout. There are some instances where the term “substance abuse disorders” is used.

• Lines 110-116: This paragraph does not really describe what that authors are referring to a “reverse causation,” it instead just reiterates the prevalence of substance use disorders in justice involved populations. I would have expected evidence for describing this pathway to include various ways in which incarceration and broader criminal legal involvement may harm mental health including studies about how solitary confinement, lengths of incarceration, violence and trauma exposures during incarceration, arrests and negative police encounters have all been associated with depression, PTSD, and other mental health symptoms.

• Lines 117-125: This paragraph refers to the reciprocal/bidirectional nature of the mental health and criminal legal involvement, which encompasses what I would have expected in the prior paragraph about “reverse causation.” Overall, the description of and distinction between the 3 pathways should be clearer.

Methods

• Clear and well-aligned with study aims.

• Statistical analysis seems adequate but reviewers with more statistical expertise should review.

Results

• The results are presented very clearly and concisely.

Discussion

• Conclusion and implications are adequately supported by the results

Tables & Figures

- Clear

Reviewer #2: This paper’s topic is an important contribution to the literature on youth legal involvement and mental health outcomes. However, framing seems to fit more in line with a criminal justice journal, the focus on youth is often lost, and the methods and presentation of results need particular refinement.

Title and Abstract:

- The paper is focused on youth but the title does not include this

- It would be helpful to include the time periods for follow up.

- The last sentence here should itself be an implication or conclusion rather than a statement that this will be discussed in the manuscript.

Language used:

- I would recommend that the authors use the terminology ‘criminal legal’ rather than ‘criminal justice’ given the lack of justice that the criminal legal system often imposes.

- There is a concerning amount of stigmatizing language used throughout, some examples are highlighted here. Please remove phrases like ‘criminal populations,’ ‘offenders,’ and ‘criminal behaviors’ as this voids the context of what in the United States is criminalized and who is seen as ‘criminal.’ It is also not person-centered language, particularly important in this work. Please say phrases such as ‘criminal legal involved individuals.’ ‘Substance abuse’ is also an outdated term and the scale used includes ‘substance use,’ so I would recommend this language or ‘substance misuse’ when referring to troubling or self-declared troubling substance use.

Introduction:

- Please rephrase that ‘research has suggested the opposite’ as both bodies of research say that heightened mental health needs and heightened criminal involvement move in the same direction. It is a question of direction of causality. However, the authors are not exploring direction of causality, so this introduction is confusing.

- Please expand on what the ‘continuum of contact’ entails from prior work.

- Define ‘institutionalization’ the first time it is used, as this often refers to institutions outside of criminal legal settings such as mental health hospitals. While ‘including facilities designed for minors’ is more specific, it is still unclear if this is state prison system facilities for youth, county-level facilities for youth, or other others.

- Clarify the following: (1) intermediate contact vs. lower-level contact like probation and (2) going to trial seems to be muddled with pre-trial jail time.

- Change ‘higher mental health issues’ to ‘higher prevalence of mental health issues.’

- I would recommend highlighting the high prevalence of legal-involved youth compared to legal-involved adults as well.

- The statement that only a small proportion of crimes are directly linked to mental illness seems too narrow. Given the co-occurrence of mental illness and substance use – often a coping strategy or self-medicating strategy – many ‘crimes’ are very closely tied to one’s mental illness. The three primary pathways described almost further this issue because they all related to substance use.

- The statement on a need for treatment programs seems out of place.

- Given the focus of the manuscript, I would recommend a criminology-focused journal. The focus on desistance, delinquency, and individual-level behavior rather than socioecological or structural factors that shape individuals’ trajectories is more aligned with criminology. If keeping here, I would recommend including the stress process model or other more structural models.

- Primary stressor isn’t necessary ‘brief and limited’ if that one sentence is quite long.

- ‘Continuum of contact’ section can easily fit with the above section and citing similar studies. For example, it is odd that Sugie and Turney’s piece isn’t cited until the later section.

- I question the focus on adult versus adolescent studies given that the focus is on adolescents. I understanding nodding to and including adult studies but it removes the context away from youth by focusing on them without the caveat throughout of how this may look different from youth.

Methods:

- Define ‘adjudicated’

- ‘Good representation of female offenders’ – please expand on this by stating the percent of female youth involved in the system versus those included in the study.

- State retention percentage across waves and how those without complete data were treated. Later, it says that only those with the outcome variable were included, but it is not clear more broadly over time.

- Move all descriptive statistics to the results section. This will also shorten the ‘control variables’ section and the individual paragraphs for each control variable can be removed. Descriptive statistics on mental health medication should be reported in conjunction with the % who qualify for mental health medication.

- If using ‘institutionalization’ then need to remove ‘incarcerated’ as the label here, as this includes secure institutions, locked facilities, and residential treatment centers. Please also present descriptive statistics (in the results) for the various forms of institutions. It is stated in the limitations that this is not available, but please say that upfront if it is not possible at all to disentangle.

- The control variables require a more thorough justification. Are they confounding variables and thus impact exposure and outcome?

o Education: is there a way to separate out high school or GED from higher education, as this seems quite distinct?

o If the individual does not have a paying job, that does not necessarily make them unemployed, as they need to be wanting and seeking work – particularly while they are still minors.

- The models allow you to assess relationships between exposure and outcome, not control variables and outcome. I am concerned by the language ‘we were interested in what sorts of social structures…’ because that implies that you are interpreting associations from control variables.

- A table comparing the final versus full sample and p-values for important differences would be helpful.

- Sensitivity analyses and a brief statement of similarity or difference of findings should be included as a supplement.

Results

- Please present confidence intervals, as they show both precision and accuracy of estimates.

- The findings are stated as ‘meaningful’ but lacking context to support this.

- Remove paragraph on associations from control variables, as the model is meant to control for the exposure-outcome relationship, not control-outcome relationship.

- I would recommend presenting the dynamic model alongside the initial model for each exposure to directly compare. Or, selecting one as the primary method and the other as a secondary analysis. It’s currently hard to follow from both. The dynamic model description also reads similarly to the first supplementary model.

- Move discussion of imputation to the methods.

Discussion:

- Emphasize focus on youth, which is one of the primary contributions.

- I would reduce the restating of results in the discussion and tone down the finality of the findings. For example, arrests may have other longer-term impacts on mental health, but other work is not cited here.

- I caution again about the comparison to other incarceration work when this outcome combines incarceration with other forms of institutionalization.

- No citations are provided for the potential benefits of incarceration.

- Remove interpretation of control variables here as well.

Implications

- The relevance to criminological theory should be moved to the discussion. However, this should also be expanded on to include public health literature on the topic that focuses less on criminological factors and more on structural and system factors.

- The practical implications should also be moved to the discussion.

- Then, the implications paragraph should more summarize the main findings, future directions, and brief implications.

**********

6. PLOS authors have the option to publish the peer review history of their article (what does this mean?). If published, this will include your full peer review and any attached files.

If you choose “no”, your identity will remain anonymous but your review may still be made public.

**Do you want your identity to be public for this peer review?** For information about this choice, including consent withdrawal, please see our Privacy Policy.

Reviewer #1: No

Reviewer #2: No

**********

[NOTE: If reviewer comments were submitted as an attachment file, they will be attached to this email and accessible via the submission site. Please log into your account, locate the manuscript record, and check for the action link "View Attachments". If this link does not appear, there are no attachment files.]

To ensure your figures meet our technical requirements, please review our figure guidelines: https://journals.plos.org/plosone/s/figures

You may also use PLOS’s free figure tool, NAAS, to help you prepare publication quality figures: https://journals.plos.org/plosone/s/figures#loc-tools-for-figure-preparation.

NAAS will assess whether your figures meet our technical requirements by comparing each figure against our figure specifications.

Attachment Submitted filename: PLOS ONE.docx

# Author comment: Author response to Decision Letter 1

**DOI:** 10.1371/journal.pone.0344895.r002

22 Dec 2025

Dear Dr. Knittel,

My co-author and I were pleased to receive your response inviting us to revise and resubmit our manuscript. Accordingly, we would like to submit the enclosed revised paper, “Effects of differential contacts with the criminal legal system on mental health outcomes of adolescents and young adults: A fixed-effects model,” Manuscript #PONE-D-25-43024, for reconsideration for publication in PLOS ONE.

We thank you and the reviewers for the time and effort in reviewing our manuscript. The feedback has been invaluable in improving the content and presentation of the paper. We have revised the manuscript according to the reviewers’ comments, and our point-by-point responses are given below (page and line numbers based on the clean manuscript). Attached are the manuscripts with track changes and a clean manuscript as requested.

Answer to comments from Editor:

1. Please ensure that your manuscript meets PLOS ONE's style requirements, including those for file naming. The PLOS ONE style templates can be found at

https://journals.plos.org/plosone/s/file?id=wjVg/PLOSOne_formatting_sample_main_body.pdf and

https://journals.plos.org/plosone/s/file?id=ba62/PLOSOne_formatting_sample_title_authors_affiliations.pdf

Answer: Edits were made according to the instructions provided.

2. Please include your full ethics statement in the ‘Methods’ section of your manuscript file. In your statement, please include the full name of the IRB or ethics committee who approved or waived your study, as well as whether or not you obtained informed written or verbal consent. If consent was waived for your study, please include this information in your statement as well.

Answer: The ethics statement can be found on page 20, line 409 and includes all the required elements.

3. We note you have included a table to which you do not refer in the text of your manuscript. Please ensure that you refer to Table 3 in your text; if accepted, production will need this reference to link the reader to the Table.

Answer: We made the edits and added this information to page 26, line 487.

4. Please include captions for your Supporting Information files at the end of your manuscript, and update any in-text citations to match accordingly. Please see our Supporting Information guidelines for more information: http://journals.plos.org/plosone/s/supporting-information.

Answer: We added a Supporting Information Captions section at the end of the manuscript (page 44, line 1031) and supplemental files were edited to comply with the journal’s requirements.

Answer to comments from Reviewer 1:

1. This was a very extensive introduction. While PLOSOne does not offer a specific word count limit, much of the background information in the introduction could be presented more concisely.

Answer: We summarized and reduced the background section as much as possible while still making the other requested edits to the manuscript.

2. Lines 90-92 “Only a very small…especially psychotic symptoms.”: As written, it is unclear whether “especially psychotic symptoms” means psychotic symptoms are more or less likely to be linked to crimes That sentence could be saying that (1) the small proportion of crimes that are linked to mental illness symptoms are usually specifically linked to psychotic symptoms, or (2) that within the small proportion of crimes linked to mental illness symptoms, an even smaller portion of crimes are linked to psychotic symptoms. Please rephrase to clarify.

Answer: To clarify this point, the sentence was removed and the paragraph edited on page 4, lines 82-88.

3. In the paragraph from lines 93-99, you introduce the 3 possible distinct pathways connecting mental health and criminal legal involvement but then the following 3 paragraphs where the authors describe each in detail are not really aligned with how they are initially introduced.

Answer: We reworked this section so that the introductory paragraph and description of the pathways match, now beginning on page 5, line 89.

4. The relationship between criminal justice contact and mental illness are complex. There is the pathway that both criminal legal contact and mental illness have common risk factors. For example, childhood trauma increases risk of both mental illness and criminal legal involvement. While I believe this might be addressed in the first pathway referring to mediating or moderating factors (“other social factors”), it is important to make this clearer if so.

Answer: This point has been clarified on page 5 beginning on line102, within the rewrite for the different pathways and in the description of control variables selected for this study beginning on page 16 line 331.

5. Also, this may just be disciplinary differences or preference but from my perspective as a clinician-scientist, mental illness includes substance use disorders and personality disorders, so when SUD and personality disorders are described as mediating/moderating factors between mental illness and crime – it is confusing. This just may be a difference between health sciences and criminology but it may be helpful to make it clear that this study considers SUD and mental illness separately.

Answer: We added a clarification of how the different types of psychiatric diagnosis are classified in our study and cited prior literature using the same/similar classifications. These edits can now be found on page 5 lines 97-102.

6. Use “substance use disorder” consistently throughout. There are some instances where the term “substance abuse disorders” is used.

Answer: This change was made throughout the paper, and we now only use the term “substance use” (or substance use disorder, where appropriate).

7. Lines 110-116: This paragraph does not really describe what that authors are referring to a “reverse causation,” it instead just reiterates the prevalence of substance use disorders in justice involved populations. I would have expected evidence for describing this pathway to include various ways in which incarceration and broader criminal legal involvement may harm mental health including studies about how solitary confinement, lengths of incarceration, violence and trauma exposures during incarceration, arrests and negative police encounters have all been associated with depression, PTSD, and other mental health symptoms.

Answer: This paragraph has been rewritten to better reflect the reverse causation element. It can now be found on page 6 lines 115-122.

8. Lines 117-125: This paragraph refers to the reciprocal/bidirectional nature of the mental health and criminal legal involvement, which encompasses what I would have expected in the prior paragraph about “reverse causation.” Overall, the description of and distinction between the 3 pathways should be clearer

Answer: Based on comments and suggestions made by the reviewer, this section of the paper was fully reworked to make these distinctions clear. The rewritten pathways section begins on page 5 and continues through to page 6.

Answer to comments from Reviewer 2:

Title and Abstract:

1. The paper is focused on youth but the title does not include this

Answer: We have now included that information in the revised title: “Effects of differential contacts with the criminal legal system on mental health outcomes of adolescents and young adults: A fixed-effects model.”

2. It would be helpful to include the time periods for follow up.

Answer: We have now added the data collection period in the abstract briefly and this information is expanded upon in more detail in the “Data and participants” part of the Methods section (page 13, lines 267-269).

3. The last sentence here should itself be an implication or conclusion rather than a statement that this will be discussed in the manuscript.

Answer: We have now added an additional sentence to the end of the abstract, summarizing our conclusions. “Thus, our paper evidences that even less severe contacts with the criminal legal system appear to be stressors that can have both short-term and long-term effects on mental health symptoms in youths and young adults.” (page 2, lines 34-36)

Language used:

4. I would recommend that the authors use the terminology ‘criminal legal’ rather than ‘criminal justice’ given the lack of justice that the criminal legal system often imposes.

Answer: We have gone through and made changes in this language throughout the paper.

5. There is a concerning amount of stigmatizing language used throughout, some examples are highlighted here. Please remove phrases like ‘criminal populations,’ ‘offenders,’ and ‘criminal behaviors’ as this voids the context of what in the United States is criminalized and who is seen as ‘criminal.’ It is also not person-centered language, particularly important in this work. Please say phrases such as ‘criminal legal involved individuals.’ ‘Substance abuse’ is also an outdated term and the scale used includes ‘substance use,’ so I would recommend this language or ‘substance misuse’ when referring to troubling or self-declared troubling substance use.

Answer: We have gone through and made changes to this language throughout paper – we no longer use any of the terms mentioned and consistently use the term substance use in the paper.

Introduction:

6. Please rephrase that ‘research has suggested the opposite’ as both bodies of research say that heightened mental health needs and heightened criminal involvement move in the same direction. It is a question of direction of causality. However, the authors are not exploring direction of causality, so this introduction is confusing.

Answer: The sentence has been rephrased based on this comment. It is relevant to clarify we are assessing causal/time ordering (not causality) in the current paper by using fixed effects modeling and lagged models (with covariates in the same wave and mental health outcomes measured at later waves).

7. Please expand on what the ‘continuum of contact’ entails from prior work.

Answer: We have now added more details about what we mean by “continuum of contact” in the Introduction (page 3, lines 54-57) better connecting it to the section on “continuum of contact” where describe prior research on this topic (page 11, line 222),

8. Define ‘institutionalization’ the first time it is used, as this often refers to institutions outside of criminal legal settings such as mental health hospitals. While ‘including facilities designed for minors’ is more specific, it is still unclear if this is state prison system facilities for youth, county-level facilities for youth, or other others.

Answer: We have now added a definition of “institutionalization” as we operationalize it in our study the first time that we mention the term in the manuscript on page 3 lines 56-57.

9. Clarify the following: (1) intermediate contact vs. lower-level contact like probation and (2) going to trial seems to be muddled with pre-trial jail time.

Answer: This wording has been removed/corrected throughout the paper to make our concepts less subjective and clearer.

10. Change ‘higher mental health issues’ to ‘higher prevalence of mental health issues.’

Answer: We have now corrected this typo on page 4 line 77.

11. I would recommend highlighting the high prevalence of legal-involved youth compared to legal-involved adults as well.

Answer: We would appreciate clarification on this point so that we can make the suggested edit. We do present percentages of mental health diagnoses for both youths and adults in the criminal legal system as compared to the general population under the section “Mental health and the criminal legal system” on pages 3-4.

12. The statement that only a small proportion of crimes are directly linked to mental illness seems too narrow. Given the co-occurrence of mental illness and substance use – often a coping strategy or self-medicating strategy – many ‘crimes’ are very closely tied to one’s mental illness. The three primary pathways described almost further this issue because they all related to substance use.

Answer: We have now rewritten this section on the three primary pathways, now beginning on page 5, line 89.

13. The statement on a need for treatment programs seems out of place.

Answer: We removed this out of place statement from the manuscript.

14. Given the focus of the manuscript, I would recommend a criminology-focused journal. The focus on desistance, delinquency, and individual-level behavior rather than socioecological or structural factors that shape individuals’ trajectories is more aligned with criminology. If keeping here, I would recommend including the stress process model or other more structural models.

Answer: We have now added a paragraph incorporating social stress and the Stress Process Model on pages 7-8. We agree this project ties in well to this literature.

15. Primary stressor isn’t necessary ‘brief and limited’ if that one sentence is quite long.

Answer: The language was edited based on this suggestion. Changes can now be seen on page 10, line 213.

16. ‘Continuum of contact’ section can easily fit with the above section and citing similar studies. For example, it is odd that Sugie and Turney’s piece isn’t cited until the later section.

Answer: We opted to keep the continuum of contact as its own separate section due to its relevance to our own paper and data analysis. That said, we did make edits to clarify links between this section and the previous section that will hopefully help the reader understand why we are presenting the narrative in this manner. Please note that we consider the Sugie and Turney piece extremely important literature and we cite it in two places before this citation (on pages 5 and 10).

17. I question the focus on adult versus adolescent studies given that the focus is on adolescents. I understanding nodding to and including adult studies but it removes the context away from youth by focusing on them without the caveat throughout of how this may look different from youth.

Answer: As our sample follows individuals from age 14 at wave 1 to age 25 at wave 11, we find the need to include both literature on teenagers and adults in this paper.

Methods:

18. Define ‘adjudicated’

Answer: We have added a definition added on page 12 line 256.

19. ‘Good representation of female offenders’ – please expand on this by stating the percent of female youth involved in the system versus those included in the study. –

Answer: We rephrased this section to more closely match what the authors reported in their seminal piece. In the original paper, the authors state “(c) a sizable enough number of female serious offenders to examine sex differences in patterns of development and desistance from crime;” (Schubert et al, 2004, p.242). Unfortunately, we do not have access to information about the number of female youth in the system during the time period and locations where the data were collected so we cannot make this comparison.

20. State retention percentage across waves and how those without complete data were treated. Later, it says that only those with the outcome variable were included, but it is not clear more broadly over time.

Answer: The retention rate for the whole study was 83%, in the final wave there were 1,134 participants who completed all 10 follow-up interviews. This information was added to page 13 lines 274-277. This was further clarified beginning on page 20, line 424.

21. Move all descriptive statistics to the results section. This will also shorten the ‘control variables’ section and the individual paragraphs for each control variable can be removed. Descriptive statistics on mental health medication should be reported in conjunction with the % who qualify for mental health medication.

Answer: We moved the descriptive statistics to the results section as requested. This section now begins on page 20, line 423. Unfortunately, information on individuals who qualify to receive medication is not available in this dataset, only information on who reports currently taking a medication for their mental health (e.g., antidepressant, antipsychotic, anti-manic)

Attachment Submitted filename: Response to reviewers.docx

# Peer review: Decision Letter 1

**DOI:** 10.1371/journal.pone.0344895.r003

**Authors:** Wing Hong Chui

27 Feb 2026

Effects of differential contacts with the criminal legal system on mental health outcomes of adolescents and young adults: A fixed-effects model

PONE-D-25-43024R1

Dear Dr. V. Oliveira,

We’re pleased to inform you that your manuscript has been judged scientifically suitable for publication and will be formally accepted for publication once it meets all outstanding technical requirements.

Within one week, you’ll receive an e-mail detailing the required amendments. When these have been addressed, you’ll receive a formal acceptance letter and your manuscript will be scheduled for publication.

An invoice will be generated when your article is formally accepted. Please note, if your institution has a publishing partnership with PLOS and your article meets the relevant criteria, all or part of your publication costs will be covered. Please make sure your user information is up-to-date by logging into Editorial Manager at Editorial Manager® and clicking the ‘Update My Information' link at the top of the page. For questions related to billing, please contact billing support.

If your institution or institutions have a press office, please notify them about your upcoming paper to help maximize its impact. If they’ll be preparing press materials, please inform our press team as soon as possible -- no later than 48 hours after receiving the formal acceptance. Your manuscript will remain under strict press embargo until 2 pm Eastern Time on the date of publication. For more information, please contact onepress@plos.org.

Kind regards,

Wing Hong Chui, PhD

Academic Editor

PLOS One

Additional Editor Comments (optional):

Reviewers' comments:

Reviewer's Responses to Questions

**Comments to the Author**

1. If the authors have adequately addressed your comments raised in a previous round of review and you feel that this manuscript is now acceptable for publication, you may indicate that here to bypass the “Comments to the Author” section, enter your conflict of interest statement in the “Confidential to Editor” section, and submit your "Accept" recommendation.

Reviewer #1: All comments have been addressed

**********

2. Is the manuscript technically sound, and do the data support the conclusions?

The manuscript must describe a technically sound piece of scientific research with data that supports the conclusions. Experiments must have been conducted rigorously, with appropriate controls, replication, and sample sizes. The conclusions must be drawn appropriately based on the data presented.

Reviewer #1: Yes

**********

3. Has the statistical analysis been performed appropriately and rigorously?

Reviewer #1: Yes

**********

4. Have the authors made all data underlying the findings in their manuscript fully available?

The PLOS Data policy requires authors to make all data underlying the findings described in their manuscript fully available without restriction, with rare exception (please refer to the Data Availability Statement in the manuscript PDF file). The data should be provided as part of the manuscript or its supporting information, or deposited to a public repository. For example, in addition to summary statistics, the data points behind means, medians and variance measures should be available. If there are restrictions on publicly sharing data—e.g. participant privacy or use of data from a third party—those must be specified.

Reviewer #1: Yes

**********

5. Is the manuscript presented in an intelligible fashion and written in standard English?

PLOS ONE does not copyedit accepted manuscripts, so the language in submitted articles must be clear, correct, and unambiguous. Any typographical or grammatical errors should be corrected at revision, so please note any specific errors here.

Reviewer #1: Yes

**********

6. Review Comments to the Author

Please use the space provided to explain your answers to the questions above. You may also include additional comments for the author, including concerns about dual publication, research ethics, or publication ethics. (Please upload your review as an attachment if it exceeds 20,000 characters)

Reviewer #1: This is a significant improvement to the initial submission. The narrative, especially in the introduction and discussion section reads much better and is well organized. The implications and conclusions are clearer. No further comments.

**********

7. PLOS authors have the option to publish the peer review history of their article (what does this mean?). If published, this will include your full peer review and any attached files.

If you choose “no”, your identity will remain anonymous but your review may still be made public.

**Do you want your identity to be public for this peer review?** For information about this choice, including consent withdrawal, please see our Privacy Policy.

Reviewer #1: No

**********

# Editor report: Acceptance letter

**DOI:** 10.1371/journal.pone.0344895.r004

**Authors:** Wing Hong Chui

PONE-D-25-43024R1

PLOS One

Dear Dr. V. Oliveira,

I'm pleased to inform you that your manuscript has been deemed suitable for publication in PLOS One. Congratulations! Your manuscript is now being handed over to our production team.

At this stage, our production department will prepare your paper for publication. This includes ensuring the following:

* All references, tables, and figures are properly cited

* All relevant supporting information is included in the manuscript submission,

* There are no issues that prevent the paper from being properly typeset

You will receive further instructions from the production team, including instructions on how to review your proof when it is ready. Please keep in mind that we are working through a large volume of accepted articles, so please give us a few days to review your paper and let you know the next and final steps.

Lastly, if your institution or institutions have a press office, please let them know about your upcoming paper now to help maximize its impact. If they'll be preparing press materials, please inform our press team within the next 48 hours. Your manuscript will remain under strict press embargo until 2 pm Eastern Time on the date of publication. For more information, please contact onepress@plos.org.

You will receive an invoice from PLOS for your publication fee after your manuscript has reached the completed accept phase. If you receive an email requesting payment before acceptance or for any other service, this may be a phishing scheme. Learn how to identify phishing emails and protect your accounts at https://explore.plos.org/phishing.

If we can help with anything else, please email us at customercare@plos.org.

Thank you for submitting your work to PLOS ONE and supporting open access.

Kind regards,

PLOS ONE Editorial Office Staff

on behalf of

Prof. Wing Hong Chui

Academic Editor

PLOS One
